# The deubiquitinase Usp27x as a novel regulator of cFLIP_L_ protein expression and sensitizer to death-receptor-induced apoptosis

**DOI:** 10.1007/s10495-021-01706-9

**Published:** 2022-01-19

**Authors:** Manuel Nico Dold, Xiulin Ng, Claudia Alber, Ian Edward Gentle, Georg Häcker, Arnim Weber

**Affiliations:** 1 Faculty of Medicine, Institute of Medical Microbiology and Hygiene, Medical Center, University of Freiburg, Freiburg, Germany; 2 BIOSS Centre for Biological Signalling Studies, University of Freiburg, Freiburg, Germany

**Keywords:** Usp27x, Apoptosis, Caspase-8, TLR3, TNF, TRIM28

## Abstract

Death receptors are transmembrane proteins that can induce the activation of caspase-8 upon ligand binding, initiating apoptosis. Recent work has highlighted the great molecular complexity of death receptor signalling, in particular through ubiquitination/deubiquitination. We have earlier defined the deubiquitinase Ubiquitin-Specific Protease 27x (Usp27x) as an enzyme capable of stabilizing the pro-apoptotic Bcl-2 family member Bim. Here, we report that enhanced expression of Usp27x in human melanoma cells leads to the loss of cellular FLICE-like inhibitory protein (cFLIP) and sensitizes to Tumor necrosis factor receptor 1 (TNF-R1) or Toll-like receptor 3 (TLR3)-induced extrinsic apoptosis through enabling enhanced processing of caspase-8. The loss of cFLIP_L_ upon overexpression of Usp27x was not due to reduced transcription, could be partially counteracted by blocking the ubiquitin proteasome system and was independent of the known cFLIP_L_ destabilizing ubiquitin E3-ligases Itch and DTX1. Instead, Usp27x interacted with the E3-ligase TRIM28 and reduced ubiquitination of TRIM28. Reduction of cFLIP_L_ protein levels by Usp27x-induction depended on TRIM28, which was also required for polyI:C-induced cell death. This work defines Usp27x as a novel regulator of cFLIP_L_ protein expression and a deubiquitinase in fine tuning death receptor signalling pathways to execute apoptosis.

**Supplementary Information:**

The online version contains supplementary material available at 10.1007/s10495-021-01706-9.

## Introduction

One of the two major apoptosis pathways starts at the cell surface with signals from death receptors such as TNFR1 and Fas/CD95; Toll-like receptor 3 (TLR3), a pattern recognition receptor that recognizes double-stranded RNA (dsRNA) and is expressed by many tumour cells, can assemble a similar death-inducing signalling complex (DISC) and trigger similar death-inducing events [1].

The DISC consists of the assembly of a number of proteins and orchestrates the activation of caspase-8. A major regulator of apoptosis-induction through death receptors (DR) is the cellular FLICE-like inhibitory protein (cFLIP) [2]. Deficiency of cFLIP leads to sensitization to DR ligand-induced apoptosis, while ectopic expression causes resistance in human melanoma cells [3]. RNAi against cFLIP further sensitizes all of a variety of researched human lung cancer cell lines to TLR3 induced apoptosis, in some cases independently of IAP-antagonists [4]. This suggests that cFLIP is a major checkpoint of TLR3-induced apoptosis and supports a model in which capase-8 recruitment to the TLR3-DISC occurs independently of RIPK1 ubiquitination.

Ubiquitination is the covalent binding of the protein ubiquitin to a substrate lysine residue, catalyzed by a substrate-specific E3-ligase. Ubiquitin itself is targeted for ubiquitination at various lysine (K) or at the N-terminal methionine (M), resulting in polyubiquitination of different linkage types that have different outcomes due to the recruitment of proteins with different ubiquitin binding domains (UBDs) [5, 6]. K63-linked ubiquitination of RIPK1 at the DISC of TNF-R1 by cIAP1/2 leads to the recruitment TAK1, formation of the linear ubiquitin chain assembly complex (LUBAC) and subsequent M1-ubiquitination of RIPK1 by LUBAC, resulting in the activation of the pro-survival NF-κΒ [7]. Depletion of NF-κΒ or inhibition of RIPK1 ubiquitination in presence of TNF results in DISC assembly and induction of apoptosis or necreptosis [7].

TLR3 can induce NF-κΒ through a complex containing TRIF, RIPK1, the TRADD/TRAF2/cIAP1/2 complex, TAK1, LUBAC and the IKK complex [8–10]. TLR3-induced apoptosis by its synthetic ligand poly-I:C (pIC) depends on TRIF, RIPK1 and caspase-8 [9], is independent of TNF [1] and negatively regulated by the TRADD/TRAF2/cIAP complex [9].

Human cFLIP has high homology with caspase-8 but is catalytically inactive. cFLIP has three protein isoforms, cFLIP_L_, cFLIP_S_ and the rarely detected cFLIP_R_ [2]. Pro-caspase-8 heterodimerization with cFLIP_S_ blocks the processing and thereby activation of caspase-8. Heterodimerization with cFLIP_L_ allows restricted caspase-8 processing, leading to the cleavage of substrates such as RIPK1 and inhibition of RIPK1 kinase-dependent apoptosis and necroptosis [2].

cFLIP-levels are known to be regulated transcriptionally by NF-κΒ [11] and by ubiquitin-dependent degradation through the E3-ligases Itch [12] and Deltex-1 (DTX1) [13]. Itch activity is positively regulated by Usp8 [14], the kinases ATM [15] and JNK [12] and negatively regulated by AKT [14, 16]. AKT itself can induce the loss of cFLIP_L_ independent of Itch or JNK [17]. Usp8 [18] and Ku70 [19] have further been reported to stabilize cFLIP_L_ through deubiquitination.

Usp27x is a deubiquitinating enzyme (DUB) capable of removing K48 and K63-linked ubiquitin from proteins *in vitro* and within cells [20, 21]. First described as suppressor of neural differentiation [22], Usp27x deficiency has been associated with X-linked intellectual disability [23]. Other reported roles include the control of histone mono-ubiquitination [24], promotion of epithelial-to-mesenchymal transition (EMT) by the stabilization of Snail1 [25], stabilization of Cyclin E (promoting growth, migration, and invasion of hepatocellular carcinoma) [26], stabilization of the cytosolic DNA-sensor cyclic GMP-AMP synthase (cGAS) [27], and the negative regulation of the cytosolic RNA-sensors RIG-1 and MDA5 [21]. Usp27x is still poorly characterized, with different protein sizes reported and no functional commercial antibody against human Usp27x available.

We have identified Usp27x as a DUB capable of deubiquitinating and stabilizing the pro-apoptotic BCL2 family member Bim in conditions of active ERK signalling, protecting it from proteasomal degradation and leading to sensitization of human cancer cells to apoptosis [20]. Interestingly, Bim deficiency only partially protected mUsp27x_s_-overexpressing cells from apoptosis through ERK-activation by Phorbol 12-myristate 13-acetate (PMA) [20]. We followed up on this observation and report here that overexpression of Usp27x in human melanoma cells leads to loss of the cFLIP_L_ protein and sensitizes to TNF and pIC induced apoptosis through enhanced processing of caspase-8. This was independent of the E3-ligases Itch and Deltex-1 but loss of cFLIP_L_ required the unrelated E3-ligase TRIM28, which is needed for pIC induced cell death.

## Materials and methods

### Cell lines, culture conditions

WM1158 and 1205Lu human metastatic melanoma cell lines (obtained from Dr. Meenhard Herlyn, Wistar Institute, Philadelphia) were cultured in TU2% melanoma medium containing 80% (v/v) MCDB153 (Sigma-Aldrich, #M7403), 20% (v/v) Leibovitz’s L-15 (Thermo Fisher Scientific (Gibco) #11415-056), 2% (v/v) FCS (Thermo Fisher Scientific, #10,270,106), 5 µg/ml insulin (Sigma: #I4011), 1.68 mM (v/v) CaCl_2_ and 1% penicillin/streptomycin (P/S, (Thermo Fisher Scientific, #15,140,130).

CaCo2 human colon carcinoma cells (provided by Tilman Brummer, Freiburg), immortalized 293FT cells (Invitrogen) and A549 human epithelial lung carcinoma cells (provided by Ulrich Maurer, Freiburg) were cultured in DMEM (Thermo Fisher Scientific, #41,965,062) supplemented with 10% FCS (Sigma-Aldrich, #F7524) and 1% P/S. Cell lines have been authenticated using DNA profiling using different and highly polymorphic short tandem repeat (STR) loci. All cells were incubated under standard culture conditions (37° C, 5% CO_2_, and 95% humidity).

Doxycycline (Dox, Sigma-Aldrich, #D9891) to induce Usp27x expression in TetR_on_ cells was used in a concentration of 1 µg/ml for the time indicated. Lentivirally transduced cells were selected using hygromycin B (Invitrogen, 293FT: 300 µg/ml; 1205Lu: 750 µg/ml and WM1158: 500 µg/ml) and/or puromycin (Invivogen, 5 µg/ml). Poly-I:C (pIC) (Sigma-Aldrich # P1530) and TNF treatment was performed as indicated. For experiments measuring active caspase-3 via FACS, FC-tagged human TNF (gift from Dr. Ian Gentle, Freiburg) was used. For IP-experiments Flag-tagged human TNF (gift from Ulrich Maurer, Freiburg) was used.

The caspase-inhibitor Q-VD-OPh (QVD) (Gentaur, #GEN1589978) was added as indicated at 10 µM concentrations. Necrostatin-1 (Nec1) (Sigma-Aldrich, #N9037) and LCL-161 (Active Biochem, #A-1147) were used as indicated. Phorbol 12-myristate 13-acetate (PMA) (Sigma-Aldrich, #P1585) was used in a concentration of 16.2 nM as indicated. Human TNF neutralizing rabbit monoclonal antibody (D1B4) (Cell Signaling, #7321) was added as indicated.

### Construction of expression vectors and generation of cell lines

Cloning of murine mUsp27x_S_ (438 aa, Q8CEG8) and generation of WM1158 and 1205Lu melanoma cells expressing GFP-mUsp27x_S_, GFP-mUsp27xC87A, or GFP-mUsp22 (all Tet_on_ systems) has been described earlier [20]. Cloning of human GFP-hUsp27x_L_ (GFP-fused to the N-terminus of full length human Usp27x_L_ (636 aa) including the published CTG start codon of human Usp27x_L_ [24]) and generation of WM1158 and 1205Lu cells expressing GFP-hUsp27x_L_ (Tet_on_) was done exactly as described for mUsp27x_S_ [20]. The same was true for cloning of 3x-Flag-hUsp27x_L_ and generation of 293FT cells inducible expressing full-length 3xFlag-hUsp27x_L_. Generation of WM1158 cells overexpressing murine Bcl-X_L_ was done using a lentiviral construct described earlier [28]. pFCMV-TO-GPI-hsTNFR2-IRES-GFP-sv40-puro was used for transient transfection to express the GPI-TNFR2 which acts as a decoy receptor for TNF [29] in 293FT cells. For overexpression of untagged human hUsp27x in 293FT cells, hUsp27x_L_ (using the described CTG start codon, 636aa) and hUsp27x_S_ (A6NNY8, using the same ATG start codon described for mUsp27x_S_, 438aa) were cloned into pENTR-SD-D-TOPO and shuffled into a pMIG-GW vector. Human ubiquitin (tagged with 6x histidine at the C-terminus) was cloned into pENTR-SD-D-TOPO and shuffled into the lentiviral vector pEF1α-GW_puro to obtain pEF1a-6xHis-hUbi_puro. Afterwards, WM1158-TetR-GFP-hUsp27x_L_ cells were infected to obtain cells stably overexpressing 6xHis-ubiquitin. All primer sequences are available on request. Production of lentiviral particles was done by transfecting 293FT cells together with packaging vectors pMD2.G and psPAX2 (Dr. Didier Trono, Lausanne) using either FugenHD (Promega) or PEI.

### Gene silencing with RNAi

Sequences of specific siRNAs were as follows: siCo3 (control and protocol see [1]); siβ-TrCP: 5′-GUGGAAUUUGUGGAACAUC-3′﻿ [20, 30]; siCaspase-8: 5′-GCUCUUCCGAAUUAAUAGA-3′﻿ [31].

### Generation of human knock out lines using CRISPR/Cas9

Gene-deficient cells were generated via CRISPR/Cas9 genome editing by transducing the cells with the lentiviral vector lentiCRISPR v2 (Addgene #52961; [32]) and selection with puromycin.

For targeting Caspase-8 or EGFP-1 same lentiCRISPR v2 was used where the puromycin resistance cassette was replaced with RFP657 for selection. The guide RNAs were: CTRL (non-targeting control from the human GeckoV2 library [28, 32]): 5′-ATCGTTTCCGCTTAACGGCG-3′; or targeting caspase-8 (two gRNAs: gCaspase-8-1: 5′-GCCTGGACTACATTCCGCAA-3′, gCaspase-8-2: 5′-GCTCTTCCGAATTAATAGAC-3′); Bax (5′-CAAGCGCATCGGGGACGAAC-3′﻿ [28]); Bak (5′-ACGGCAGCTCGCCATCATCG-3′﻿ [28] (caspase-8 and Bax/Bak gRNAs were designed using the MIT server (http://crispr.mit.edu/), now discontinued). EGFP-1 gRNA: 5′-GGGCGAGGAGCTGTTCACCG-3′﻿ [33]. The following gRNAs were taken from the Brunello human gRNA database [34]: TRIM28-E3 (targets exon#3): 5′-CCAGCGGGTGAAGTACACCA′-3′; Usp27x-2: 5′-TAAACCGATCGTAAAGCTGG-3′; Usp27x-3: 5′-CGGGACTCGGCATCTCACAT-3′; Itch-E8 (targets exon#8): 5′-AATACAAACCTGGTCTACGT-3′; Itch-E11 (targets exon#11): 5′- GAACGGCGGGTTGACAACAT-3′; DTX1-E1 (targets exon#1): 5′-GTGCTGAAGGAGGACGCTCG-3′; DTX1-E2 (targets exon#2): 5′-GTGTGGGAGTGGGAGAACGA-3′.

Generation and validation of a single Usp27xKO clone in 293FT (clone 2/10) was described earlier [20] and CaCo2 (clone 1/5) cells were generated in parallel using the same gRNA and vector systems (Usp27x-4; 5′-GTGAGATGTCGTCGCTGTTT-3′).

### Detection of cell death

Cell lines were seeded in 6-well plates (1 × 10^5^ cells for WM1158; 1.5 × 10^5^ cells for 1205Lu, 2.5 × 10^5^ for 293FT) and stimulated as indicated in the figures. Cells were washed in PBS, fixed in 4% formalin, permeabilized with 0.5% saponin (Roth #4185.1) and stained for 30 min with anti-active caspase-3 antibody (BD: #559565 dilution 1:500) or with an antibody specific for active Bax (6A7 clone, Sigma #B8429, dilution 1:300), followed by 30 min incubation with secondary antibody (donkey anti-rabbit IgG (H+L) AlexaFluor 647; Dianova: 711-605-152; or donkey anti-mouse IgG (H+L) Cy5; Dianova: 715-175-151; 1:500 dilution in PBS/0.5% BSA/0.5% Saponin). Flow cytometry was performed using a FACS Calibur (Becton Dickinson). In some experiments harvested cells were supplemented with propidium iodide (Sigma, P4170, 5 µg/ml) immediately prior to analysis by flow cytometry.

#### ELISA

Cells were seeded in 6-well plates as described. Supernatants of stimulated cells were collected and soluble fraction was obtained by centrifugation. Human TNF ELISA was performed according to the manufactures protocol (ELISA MAX™ Deluxe Set Human TNF-α, BioLegend: #430204) on uncoated 96 Nunc™ MaxiSorp™ ELISA Plates (BioLegend: #423501) in technical triplicates per biological sample. The range of the standard was modified from 7.8 to 500 to 1,953125–500 pg/ml. The samples were then measured by a Tecan ELISA-Reader with Magellan software. Absorbance at 450 nm was measured and the absorbance at 570 nm was subtracted from the one at 450 nm. The standard curve was plotted on a log–log axis graph with concentration on the x-axis and absorbance on the y-axis. Standard curve-fitting was handled by Magellan using a 4-parameter curve-fitting. Concentrations of samples were calculated based upon the standard curve.

### Fluorescence microscopy

1205Lu and WM1158 inducible expressing murine or human GFP-Usp27x were stimulated for 24 h with doxycycline and analyzed with a BZ 9000E microscope (Keyence) and photos were processed using the BZ II Analyzer software 1.42 (Keyence).

### Quantitative polymerase chain reaction (qPCR)

1 × 10^6^ TetR-GFP-hUsp27x_L_ melanoma cells were seeded in a 10 cm dish (10 ml media) for inducing gene expression the day after. RNA was isolated using High Pure RNA Isolation Kit (Roche, #11828665001) according to the manufactures protocol and RNA concentrations were measured using NanoDrop. 1 µg of RNA per sample was transcribed into cDNA using Transcriptor First Strand cDNA Synthesis Kit (Roche, #04897030001) according to the manufactures protocol using anchored-oligo(dT)_18_ Primer. qPCR was performed using LightCycler® TaqMan® Master (Roche, #04735536001) and the Universal ProbeLibrary Set, Human (Roche, #4683633001; Assay Designer: https://lifescience.roche.com/en_de/brands/ universal-probe-library.html; both discontinued) on a ThermoFisher LightCycler II carrousel-based system using LightCycler Capillaries (Roche, #04929292001) according to the manufactures protocol with the following Mix/Primers and probes: per sample 0.4 µl of forward/reverse primers [20 µM stock], 0.4 µl UPL-probe was mixed with 9.8 µl DNAse free water and 4 µl of Master (Roche TaqMan) was added. Target genes were: cFLIP_L_ (fwd primer: 5′-gctcaccatccctgtacctg-3′; rev primer: 5′-caggagtgggcgttttctt-3′; UPL probe: UPL14); HPRT (fwd primer: 5′-tgaccttgatttattttgcatacc-3′; rev primer: 5′-cgagcaagacgttcagtcct-3′; UPL probe: UPL73); GAPDH (fwd primer: 5′-agccacatcgctcagacac-3′; rev primer: 5′-gcccaatacgaccaaatcc-3′; UPL probe: UPL60). 15 µl of qPCR Master-Mix was pipetted into the capillaries. On top 5 µl of diluted cDNA was added and the capillaries were centrifuged at 700 g in a table-top centrifuge. cDNA dilutions were 1:10 (5 µl of 1:10 resembles 25 ng of RNA/transcribed cDNA) in case of experiments looking for cFLIP_L_ expressions. Technical duplicates reactions (of three individual performed experiments) were measured and analyzed using LightCycler 2.0 carousel-based system and software (Roche). Setup of the qPCR machine occurred according to Roche: “LightCycler TaqMan Master (Version 9)” manual with 45 quantification cycles.

### Analysis of quantitative polymerase chain reaction (qPCR)

Of the measured raw Cp-values the average “mean Cp” of the technical replicates were calculated. Of the “mean Cp” of the control group which should be normalized to the “average control Cp” was determined by averaging the “Mean Cp” of each reference and target gene. ΔCp-vales for each sample where then calculated by subtracting the “Mean Cp” of the respective “average control Cp”.

$${\Delta }\text{C}\text{p}=average\, control \,Cp-Mean \,Cp$$

Next, the relative quantity (RQ) of each sample was calculated with help of the reaction efficiencies (RE). Reaction efficiencies (REs) were determined before analysis by creating a standard curve for each primer pair.

$$RQ=RE^{\Delta\text{C}\text{p}}$$

RE was the following: cFLIP_L_: 2.048; HPRT: 1.906; GAPDH 1.909.

For cFLIP_L_ qPCR experiments a normalization factor for each sample was calculated by taking the geometric mean of RQ (reference gene 1) and RQ (reference gene 1):

$$NF \,of \,sample = geomean\,[RQ\left(reference \,gene\,1\right)+RQ\left(reference\, gene \,2\right)]$$

The normalized expression of the sample was then calculated the following way:

$$normalized \,expression \,of sample\,=\frac{RQ\left(target \,gene\right)}{NF}$$

The normalized expressions of the samples were imported into Graph Pad Prism 8 to create the summarized graphs by visualizing the geometric mean of each biological group together with the geometric standard deviation (SD).

### Western blotting (WB)

**Fig. 1:**
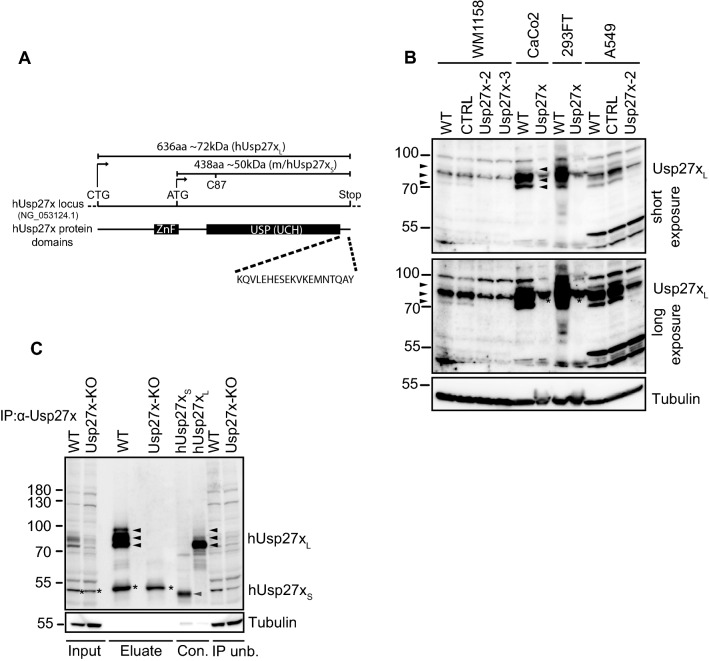
Detection of endogenous human Usp27x. **A**, Schematic representation of the human Usp27x locus, Usp27x protein domains and the used human and mouse Usp27x variants. The human Usp27x locus (hUsp27x, NG_053124.1) is shown with the predicted/annotated ATG start codon which results in a 438 amino acid (aa) human Usp27x (named hUsp27x_S_ in this study) protein variant (A6NNY8, NCBI RefSeq.: NP_001138545.1). Of note, the murine Usp27x is predicted to also start with an ATG resulting in an Usp27x variant of the same size and domain structure (438aa, named mUsp27x_S_ in this study; Q8CEG8, NCBI RefSeq.: NP_062334.2). CTG indicates the alternative discovered start site [24] of human Usp27x which results in a protein with an 198aa extension at the N-terminus encoding a ZnF domain, absent in Usp27x_S_. This human Usp27x variant (named hUsp27x_L_ in this study) is predicted to encode a protein of ~72 kDa (636 aa, [24]). Both Usp27x variants share the catalytic USP domain containing the UCH. The critical cysteine at position 87 (C87 in h/mUsp27x_S_) for the active site corresponds to the position C285 in hUsp27x_L_ [20]. The last 19 aa (KQVLEHESEKVKEMNTQAY) present in both human Usp27x variants were used in this study as a target peptide sequence for the generation of the custom-made hUsp27x-antibody. **B**, Detection of endogenous human Usp27x by Western blot with the custom-made hUsp27x antibody confirms a variant of Usp27x that is larger than the predicted annotated form in various tested human cell lines. Endogenous human Usp27x of whole-cell-lysates from wild-type (WT), control (CTRL) or polyclonal Usp27x-deficient (Usp27x-2 and Usp27x-3) WM1158 melanoma or A549 lung cancer cells, as well as WT or single clone Usp27x-deficient CaCo2 (Usp27x-deficient clone 1/5) or 293FT cells (Usp27x-deficient clone 2/10) were detected by custom-made Usp27x-antibody via Western blotting. Arrow-heads indicate the specific signal for human Usp27x, asterisks indicate unspecific signal (n = 3). **C**, The custom-made hUsp27x-antibody is able to detect hUsp27x_S_, but only detects hUsp27x_L_ at the endogenous level by Western blotting and immunoprecipitation. Endogenous human Usp27x from WT and single clone (clone 1/5) Usp27x deficient (Usp27x-KO) CaCo2 colon carcinoma lysate (800 µg) was immunoprecipitated with 4 µg of the custom-made hUsp27x antibody. The eluate was blotted along with 100 µg input, 100 µg IP-unbound flow through faction (IP unb.) and 10 µg lysate from 293FT cells transiently transfected with hUsp27x_S_ and 5 µg lysate from 293FT cells transiently transfected with hUsp27x_L_ (Con.). Black arrow-heads indicate specific signal for hUsp27x_L_, grey arrow-heads indicate expected signal of the predicted hUsp27x_S_, asterisks indicate unspecific signal in input and eluate (n = 3 for the IP, n = 2 for transient transfected controls)

Harvested cells were lysed in DISC lysis buffer (20 mM Tris/HCl, pH 7.4, 150 mM NaCl, 10% glycerol, 1% Triton X-100 supplemented with 1 × Protease-Inhibitor (Roche), 1x Phospho-Stop (Roche), 25 mM NEM, 5 mM EDTA, 5 mM 1,10-Phenanthroline, 75 µM PR-619 (Selleck), 20 µM MG132), as in Fig. 3D or in immunoprecipitation experiments (Figs. 1C, 5 and Supplementary Fig. S1A S6B). In some experiments harvested cells were lysed in 6 M urea, 20 mM Tris-HCL (pH 7.5), 135 mM NaCl, 1.5 mM MgCl_2_ supplemented with 1 × Protease-Inhibitor (Roche), 1 × Phospho-Stop (Roche), 25 mM NEM, 5 mM EDTA, 5 mM 1,10-Phenanthroline, 75 µM PR-619 (Selleck), 20 µM MG132, as in Fig. 4A–C, F–G, Supplementary Fig. S4, S6A, S7A or directly in 1 × Laemmli buffer (Figs. 1B, 4E and 6A, D, F, Supplementary Fig. S1B, S2B).

Benzonase (Merck # 70746) was added in case of DISC or urea lysis and protein concentration was analyzed by Bradford (Bio-Rad Protein Assay Dye Reagent Concentrate, 450 ml #5000006) according to the manufactures protocol. As last step, WB-samples were heated (95 °C) for 5 min and loaded on Tris-Glycine 4-20% gradient gels (Thermo Fisher Scientific). Proteins were transferred to 0.2 µM PVDF membranes by wet transfer, and specific proteins were detected with the following primary antibodies: β-Actin (Sigma, A5441), Bak (Millipore, #06-536), DTX1/DTX4 (R&D systems, MAB7157), FADD (BD, #556402), FLIP (Enzo, ALX-804-961-0100), GAPDH (Millipore, MAB374), KU70 (Santa Cruz, sc-5309), phosphor IRF3 (Ser386, Abcam, #76493), RIPK1 (BD, #610459), TRAF6 (R&D systems, AF3284), Tubulin (Sigma, T9026), USP8 (R&D systems, AF7735). From Cell Signaling (CST) the following antibodies were used: Bax (#2774), Bcl-X_L_ (#2764), Bim (#2933), caspase-8 (#9746), CHIP (#2080), cIAP1 (#7065), cIAP2 (#3130), c-Jun (#9165), GFP (#2956), Itch (#12117), phospho IkBα (#9246), phospho JNK (#4668), phospho Akt (Ser473, #4058), phospho Akt (Thr308, #2965), phospho TAK1 (#4508), phospho TBK1/NAK (#5483), RIPK1 (#3493), TBK1/NAK (#3504), β-TrCP (#4394), TRAF2 (#4724), TRAF3 (#4729), TRIF (#4596), TRIM28/KAP1/TIF1beta (#4124), pTRIM28 (Ser824, #4127). Secondary antibodies used were goat-anti-rabbit (Sigma, A6667), goat-anti-mouse (Dianova, 115-035-166), mouse anti-goat (Dianova, 205-035-108) and Donkey anti-sheep (Dianova, 713-035-003), coupled to peroxidase. Signals were detected with ECL Pico/Prime/Femto using a chemiluminescence detection system (Intas Detection Systems). If necessary stripping of the membrane prior to re-probing was performed as described in [35].

### Immunoprecipitation

For IP, harvested cells were lysed in DISC lysis buffer as described earlier. 30 µl anti-FLAG M2 affinity gel (Sigma-Aldrich, #A2220) or protein G agarose (Millipore Sigma, #11719416001) was collected and washed with 20 ml DISC wash buffer (DISC lysis buffer without any addition of supplements). Protein G agarose was coupled with either 2 µg caspase-8 p18 antibody (Santa Cruz, sc-6136), 2 µg GFP antibody (Roche, 11814460001), custom-made human Usp27x (2-4 µg, see below), mouse mAb IgG1 isotype control antibody (Cell Signaling, #5415) or rabbit mAb IgG isotype control antibody (Cell Signaling #3900). Lysates were added as indicated and were incubated for 4 h at 4 °C on a roller. Columns were washed 2x with 20 ml DISC wash buffer and elution was performed with 3x Laemmli (5 min. 95 °C). Eluted samples were loaded alongside input (whole cell lysates) on SDS-gels as described earlier.

Heavy chain specific goat anti-rabbit (Millipore, AP156P) and goat anti-mouse (Millipore, AP127P) or light chain specific rabbit anti-mouse (Cell Signaling, 58802) and mouse anti-rabbit (Jackson I.R. Lab., 211-032-171) peroxidase coupled secondary antibodies were used for detection to avoid cross reaction with the IP-antibody.

### Generation of hUsp27x-antibody

The custom-made rabbit anti-human Usp27x polyclonal antibody (appropriate applications tested: Western blot and immunoprecipitation) was produced at Thermo Fisher Scientific by immunizing two rabbits (days 14, 42, 56, 132) with a KLH-conjugated purified peptide corresponding to the last 19 amino acids (KQVLEHESEKVKEMNTQAY) of the annotated human hUsp27x_S_ (A6NNY8). Blood samples were collected on days 28, 56, 72, and 148. On day 148, antibody was affinity purified from 25 ml of serum from one rabbit (AB0306) utilizing the same peptide used for immunization. The antibody concentration obtained was 1.63 mg/ml (the ELISA titer tested by Thermo Fisher Scientific was 20 ng/ml). The antibody was stored at -20 °C in 50% glycerol at 0.815 mg/ml.

### Purification of His-ubiquitin proteins from WM1158 His-ubiquitin-TetR-GFP-hUsp27x_L_ cells

1 × 10^6^ WM1158 His-ubiquitin TetR-GFP-hUsp27x_L_ cells were seeded for 4-5 days in a 15 cm dish (two dishes per sample). The medium was changed once on the day after seeding and once on the day of stimulation. Cells were stimulated 4 h with Dox to induce hUsp27x_L_. After 2 h of induction 50 ng/ml pIC was added for the remaining 2 h. His-ubiquitin-labelled proteins (1-2.5 mg) were purified by Ni^2+^-NTA affinity chromatography as described [20].

### Data analysis and statistical analysis

FACS data were analyzed using FlowJo 10. Relative quantification of the shown immunoblots was done using the LabImage1D (INTAS) software. The analyzed data were imported into Graph Pad Prism 8 for statistical analysis and creation of graphs. Statistical significance was determined by performing a two-tailed student’s t-test. A p-value or adjusted p-value (correction for multiple comparisons using the Holm-Sidak method) was considered statistically significant p < 0.05 (*p < 0.05; **p < 0.005; and ***p < 0.0005). Routinely, three biological replicates were performed if not otherwise stated.

## Results

### A Usp27x-specific antibody detects endogenous Usp27x and confirms expression of one variant of human Usp27x

Using custom-made Usp27x antibodies, Atanassov et al. reported a size of human and mouse Usp27x of ~72 kDa, which differs from the estimated size of 50 kDa of annotated Usp27x [24]. A number of other reports, using commercially available antibodies [26, 27, 36], likewise have reported the smaller form of Usp27x, while one study failed to detect Usp27x with these antibodies despite a biological effect of the protein [21]. It has been suggested that Usp27x uses a non-canonical start site with a CTG-start codon upstream of the annotated ATG start site, leading to the expression of the 72 kDa protein (here referred to as Usp27x_L_). Compared to the annotated protein (‘Usp27x_S_’), Usp27x_L_ contains an additional N-terminal 198 amino acids encoding a UBP-type zinc finger (ZnF-UBP); such a domain is also present in the highly homologous proteins Usp22 and Usp51 [24] (see Fig. 1A for Usp27x variants).

We generated an Usp27x specific antibody by immunization of rabbits with a C-terminal epitope of human Usp27x (Fig. 1A) and affinity purification with the same peptide epitope. This antibody detected endogenous Usp27x with a size of ~72 kDa in several cell lines, and careful analysis showed three distinct bands of human Usp27x of ~72-85 kDa (Fig. 1B, C). The analysis of polyclonal Usp27x-deficient (WM1158 and A549) and monoclonal Usp27x-deficient cell lines (293FT and Caco2 cells) by Western blot as well as immunoprecipitation experiments identified all three bands as Usp27x. For the medium-size Usp27x isoform, the antibody picked up a signal of an apparently cross-reactive protein of very similar size. This band was less pronounced in cells genomically deficient for Usp27x (Fig. 1B, C), and this protein was not immunoprecipitated under native conditions from Usp27x-deficient Caco2 cells (Fig. 1C).

Of note, three bands of the same size as endogenous Usp27x were observed when Usp27x_L_ was experimentally over-expressed in 293FT cells although the relative abundance was different (compare lane 3 & 6, Fig. 1C). The origin of these three forms of Usp27x_L_ is unclear but Usp27x can be modified by posttranslational modifications like ubiquitination and phosphorylation (see below, Fig. 7 and [37]).

Importantly, Usp27x_L_ (~72 kDa) was detected in all cells tested although the expression levels varied substantially. Neither by Western blot nor by immunoprecipitation from different cell lines (Fig. 1B, C and also 1205Lu melanoma cells, HCC827 lung carcinoma, K562 chronic myelogenous leukemia and MDA-MB231 breast cancer cells; data not shown) we were able to detect a signal for Usp27x_S_ (around 50 kDa). Nevertheless, it is still possible that a 50 kDa Usp27x_S_ protein is only expressed in certain human cell types not tested here or only under certain conditions. We therefore compared both hUsp27x_L_ (human) and mUsp27x_S_ (mouse) for their biological activity.

### Usp27x_L_ and Usp27x_S_ show similar sub-cellular localization and ability to bind to and stabilize Bim

When experimentally expressed, both isoforms had the same biological activity towards Snail [25]. We compared the Bim-stabilizing activity of hUsp27x_L_ and mUsp27x_S_. When inducibly expressed in phorbol 12-myristate 13-acetate (PMA)-stimulated 293FT cells, both isoforms (mUsp27x_S_ was already published earlier [20]), interacted with endogenous Bim and protected it from PMA-induced degradation (Supplementary Fig. S1A). Interestingly, we observed binding of both Usp27x isoforms to RIPK1 (Supplementary Fig S1A, see below). As previously published for mUsp27x_S_, the large hUsp27x_L_ protein could also stabilize Bim in the BRAF-V600E positive WM1158 melanoma cell line (Supplementary Fig. S1B [20]). Finally, we compared the subcellular localization of Usp27x variants by fusing it to GFP. Upon induction in two melanoma cell lines stably carrying either a GFP-Usp27x_S_ or a GFP-Usp27x_L_, GFP fluorescence was found both in the nucleus and the cytosol for both proteins (Supplementary Fig. S1C). Of note, the GFP-Usp27x_L_ variant seemed to be more dominantly located in the nucleus, consistent with earlier reports of its localization [24], [25]. Usp27x may therefore have a nuclear function [24] but appears also to target nonnuclear proteins.

### Usp27x sensitizes 293FT cells to apoptosis through a contribution from an autocrine TNF loop that involves caspase-8 activation when stimulated with PMA

**Fig. 2:**
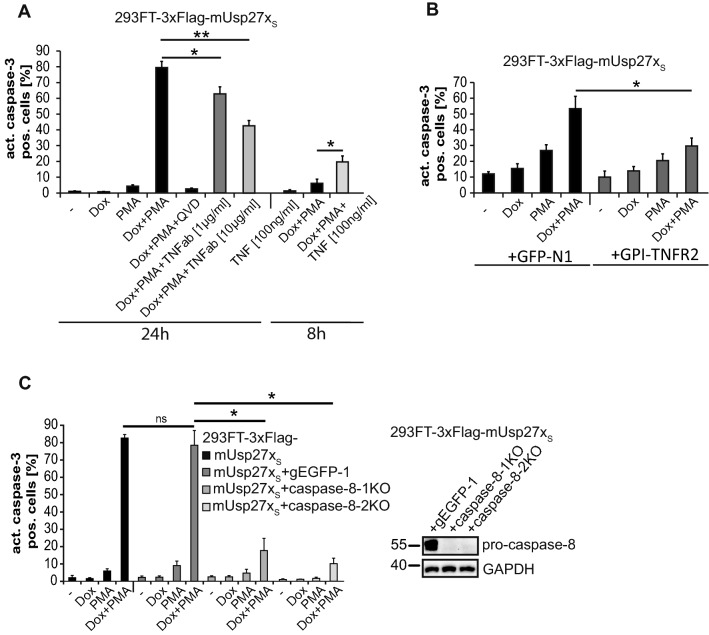
Usp27x expression sensitizes 293FT cells to apoptosis through a contribution from an autocrine TNF loop that involves caspase-8 activation when stimulated with PMA. **A**, A TNF neutralizing antibody (TNFab) blocks Dox + PMA induced apoptosis in 293FT overexpressing mUsp27x_S_ cells while exogenous TNF enhances apoptosis. Cells were seeded and treated the next day for 24 h or 8 h as indicated. TNFab was added 30 min before stimulation with Dox + PMA (n = 4). **B**, Same cells transiently transfected with a decoy receptor for TNF that contains only the ectodomain of TNFR2 (GPI-TNFR2, control: GFP-N1) show reduced apoptosis when treated with Dox + PMA (24 h, n = 3). **C**, Caspase-8 deficiency blocks apoptosis. 293FT-TetR-3xFlag-mUsp27x_S_ or two different polyclonal derivatives where the caspase-8 locus had been targeted by CRISPR/Cas9 (control: non targeting gEGFP-1, the knock-out efficiency is shown in the figure to the right), were treated as indicated for 24 h. Apoptosis was measured after 24 h of treatment by staining for active caspase-3, followed by flow cytometric analysis (n = 3). **A**–**C**, Data (means, ns: not significant (adjusted p value > 0.05)). Error bars represent SEM. *, **: significant, see 2 section

As reported earlier, expression of Usp27x sensitized 293FT cells to apoptosis induction when stimulated with PMA (Fig. 2A [20]). Although this apoptosis-induction showed dependency on Bim, Bim-deficient cells were still sensitized to Usp27x_S_-expression in the presence of PMA [20]. PMA can trigger NF-κB activation [38] to stimulate TNF production, which in turn may activate the death receptor signalling pathway. Indeed, 293FT cells expressing ectopic murine Usp27x_S_ secreted substantial amounts of TNF (Supplementary Fig. S2A) and showed activation of pro-caspase-8 when treated with PMA (Supplementary Fig. S2B). PMA-treatment was paralleled by reduction in the levels of the NF-κB-inhibitor IκBα. IκBα-degradation is achieved through the activity of the E3-ubiquitin ligase complex SCF^β-TRCP^ [39], and it has been reported that Usp27x can bind β-TrCP [20, 25]. RNAi against β-TrCP [20] or caspase-8 reduced apoptosis induction in Usp27x-expressing, PMA-treated cells (Supplementary Fig. S2C). Next, we tested whether TNF production/signalling was involved in cell death induced by mUsp27x_S_ plus PMA. Indeed, a TNF-neutralizing antibody added to the media blocked apoptosis-induction by Usp27x-expression/PMA by about 50% (Fig. 2A). When cells were transfected with a construct encoding GPI-TNFR2 (which acts as a plasma membrane-anchored decoy receptor for TNF that contains only the ectodomain of TNFR2 and can thus bind to TNF but cannot transmit signals), apoptosis was also reduced (Fig. 2B, [29]). Further, exogenous TNF was able to induce apoptosis in Usp27x-expressing, PMA-stimulated cells, suggesting that this treatment provided sensitization (Fig. 2A, 8 h). We generated two polyclonal caspase-8 deficient cell lines using two different gRNAs. Both caspase-8 deficient 293FT cell lines were nearly completely protected against apoptosis triggered by PMA plus Usp27x expression (Fig. 2C).

These results suggest that during PMA-stimulation, an autocrine TNF loop contributes to Usp27x-dependent apoptosis via the TNF receptor-mediated extrinsic apoptosis pathway by activating caspase-8 and subsequently caspase-3 in 293FT cells.

### Overexpression of Usp27x sensitizes 1205Lu and WM1158 melanoma cells to TNF and TLR3-ligand polyI:C via the extrinsic apoptosis pathway independently of RIPK1 kinase activity and cIAPs

Induction of mUsp27x_S_ and treatment with PMA triggered processing of caspase-8 and apoptosis in 293FT cells, likely supported by a TNF-autocrine/paracrine loop. TLR3-stimulation with polyI:C (pIC) can also be a pro-apoptotic stimulus. We have characterized a TLR3-dependent pathway of apoptosis induction in melanoma cells that requires the adapter TRIF and uses similar signalling to TNF-R1, involving the activation of caspase-8 controlled by cIAP1/2 [1]. We used this melanoma cell model to test whether expression of Usp27x can also control apoptosis-induction through TLR3.

To compare the biological activity of human and mouse Usp27x isoforms during TNF/pIC treatment, we generated 1205Lu and WM1158 melanoma cells inducibly expressing either mUsp27x_S_ or hUsp27x_L_. As controls, cells were generated to express mUsp27x_S_-C87A (a catalytically inactive mUsp27x [20]) or the closest homologue of mUsp27x, mUsp22 [24].

**Fig. 3:**
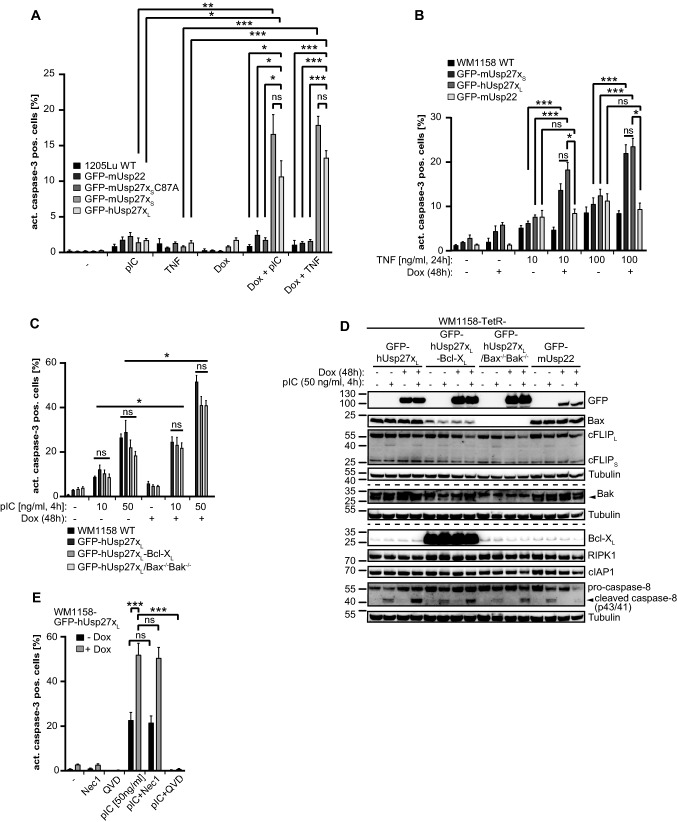
hUsp27x_L_ or mUsp27x_S_ overexpression sensitizes 1205Lu and WM1158 human melanoma cells to TNF- and pIC induced apoptosis.  **A**, Overexpression of enzymatic active mUsp27x_S_ or hUsp27x_L_ specifically sensitizes 1205Lu to TNF and pIC induced apoptosis. Wilde Type (WT), mUsp22, Usp27x_S/L_-inducible (mUsp27x_S_-C87A is catalytically inactive [20]) 1205Lu cells were treated 72 h with doxycycline (Dox) and/or 100 ng/ml TNF / 50 µg/ml poly-I:C (pIC). Active caspase-3 as a marker for apoptosis was determined by flow cytometry. Shown is the rate of active-caspase-3 positive cells (n = 5). **B**, Induction of mUsp27x_S_ or hUsp27x_L_ enhances apoptosis in TNF treated WM1158 cells in a dose dependent manner. WT or inducible WM1158 cells were pre-stimulated 24 h with doxycycline to induce Usp27x or Usp22 and afterwards indicated concentrations of TNF were added for additional 24 h. Active caspase-3 was determined by flow cytometry (WT, n = 7; TetR-GFP-mUsp27x_S_, n = 8; TetR-GFP-hUsp27x_L_, n = 5; TetR-GFP-mUsp22, n = 3). **C**, Induction of hUsp27x_L_ enhances apoptosis in pIC treated WM1158 cells in a dose dependent manner independently of the mitochondrial pathway. GFP-hUsp27x_L_-inducible WM1158, Bax^−/−^/Bak^−/−^ GFP-hUsp27x_L_- inducible WM1158 or constitutional overexpressing Bcl-X_L_ GFP-hUsp27x_L_-inducible WM1158 cells were pre-stimulated 48 h with Dox to induce Usp27x and afterwards indicated concentrations of pIC were added for 4 h. Again, active caspase-3 was determined by flow cytometry (TetR-GFP-hUsp27x_L_ treated with 50/ 100 ng/ml pIC, n = 3; other samples: n = 4). **D**, The mitochondrial pathway does not restrict caspase-8 processing or cFLIP_L_ decrease in hUsp27x_L_ overexpressing and pIC treated WM1158 cells. DISC-lysates of indicated doxycycline (Dox) inducible WM1158 cells. Cells were or were not pre-treated 48 h with Dox and then pIC was added to indicated samples for 4 h. Proteins were detected by Western blot. Arrow-heads indicate specific signal (n = 1). **E**, pIC-induced apoptotic cell death in Usp27x over-expressing WM1158 melanoma cells is not dependent on RIPK1 kinase activity. GFP-hUsp27x_L_ inducible WM1158 cells were pre-stimulated 48 h with Dox. Cells were then pre-treated 30 min with Nec1 [10 µM] to inhibit RIPK1 kinase activity. Afterwards 50 ng/ml pIC was added for 4 h. QVD [10 µM] was added as indicated. Active caspase-3 was determined by flow cytometry (n = 4). **A-C, E**, Data (means, ns: not significant (adjusted p value > 0.05)). Error bars represent SEM. *, **, ***: significant, adjusted p values, see 2 section

Wild-type 1205Lu, 1205Lu inducibly expressing mUsp27x_S_-C87A or mUsp22 showed little cell death when stimulated with TNF or pIC. TNF and pIC induced significant caspase-3 processing and cell death only in cells overexpressing hUsp27x_L_ or mUsp27x_S_ (Fig. 3A, Supplementary Fig. S3A). Cell death was blocked by caspase- but not RIPK1-inhibition (Supplementary Fig. S3A), and caspase-3-activation as well as Bax-activation was inhibited by the over-expression of Bcl-X_L_ (Supplementary Fig. S3B, S3C). In pIC treated conditions, only minute amounts of TNF were secreted, making an autocrine TNF loop unlikely for pIC/Usp27x-induced apoptosis (Supplementary Fig. S3D). Deletion of caspase-8 prevented apoptosis induced by TNF/Usp27x (Supplementary Fig. S3E). These results show that over-expression of Usp27x can sensitize human cells to apoptosis induced by both TNF and TLR3 and mediated by caspase-8.

Similar results were obtained with WM1158 melanoma cells. TNF treatment alone induced little apoptosis, as did overexpression of either form of Usp27x alone. These cells are very sensitive to pIC, and relatively low doses of pIC did induce some apoptosis. Overexpression of hUsp27x_L_ or mUsp27x_S_ increased the apoptosis rate in TNF and in pIC-treated cells (Fig. 3B, C). As expected, the mUsp27x homolog mUsp22 showed no effect on TNF-induced apoptotic cell death (Fig. 3B), and again inhibition of caspases but not RIPK1 inhibited apoptosis (Fig. 3E). Unlike the findings in 1205Lu cells, expression of Bcl-X_L_ or co-deletion of Bax and Bak (Fig. 3D) did not significantly reduce apoptosis (Fig. 3C). Thus, in WM1158 cells, pIC signals through caspase-8 in a way not requiring mitochondria (‘type I’ cells, [40]), again demonstrating that the way of action of Usp27x does not involve Bim.

**Fig. 4:**
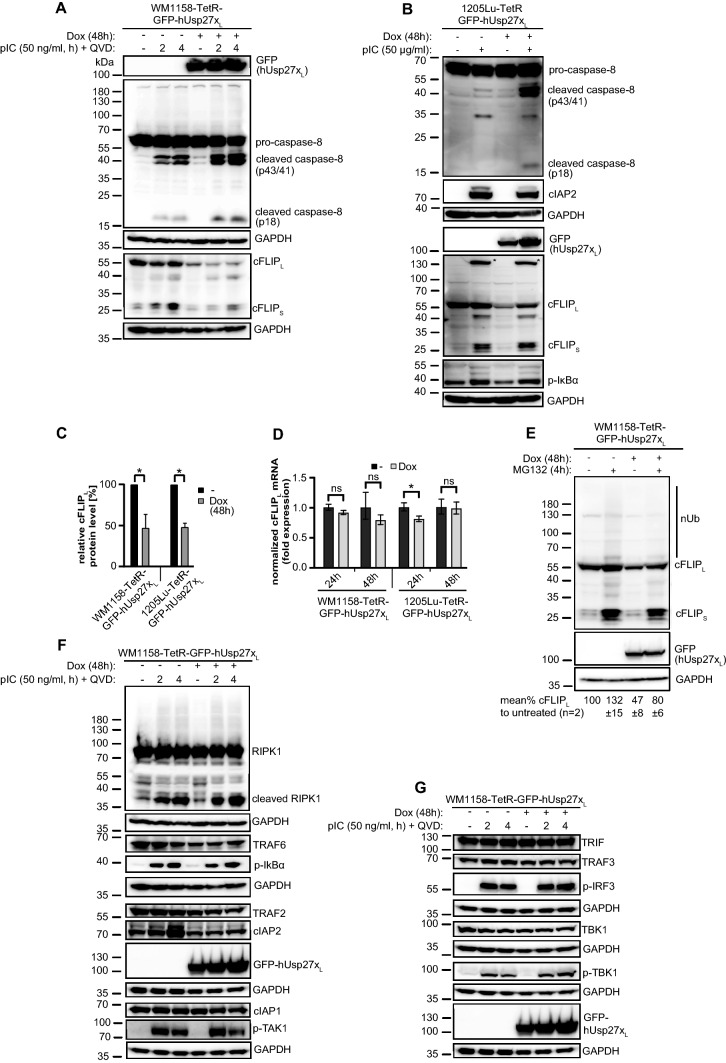
Induction of hUsp27x_L_ in WM1158 and 1205Lu leads to loss of cFLIP_L_ protein and processing of caspase-8 when treated with pIC, but does not alter NF-κB or type I interferon signalling **A**, **B** hUsp27x_L_ induction decreases cFLIP_L_ protein levels and increases caspase-8 processing upon pIC treatment. GFP-hUsp27x_L_ doxycycline-inducible WM1158 (**A**) were pre-stimulated 48 h ± Dox to induce Usp27x_L_ and afterwards QVD [10 µM] ± pIC was added for the indicated time points. 1205Lu cells inducibly expressing GFP-hUsp27x_L_ (**B**) were stimulated for 48 h ± Dox, ± pIC at the same time as indicated. Cells were lysed in urea lysis buffer and proteins were analyzed by Western blot (for **A, B**: n = 3). **C** Quantification of cFLIP_L_ protein levels detected by Western blotting (**A, B**) after 48 h dox treatment normalized to GAPDH. Shown are the cFLIP_L_ protein levels relative to the untreated control (*: p value < 0.05, error bars represent SEM; n = 3). **D** Overexpression of hUsp27x_L_ does not alter cFLIP_L_ mRNA level. GFP-hUsp27x_L_ doxycycline-inducible WM1158 and 1205Lu cells were treated or not with Dox the day after seeding and harvested at the indicated time points. The mRNA levels of cFLIP_L_ and house-keeping genes (HPRT and GAPDH) were measured by qPCR. Obtained values were normalized to the respective uninduced cell-line. Error bars represent the geometric SD (ns: adjusted p value > 0.05); *: statistically significant = p-value < 0.05 (n = 3). **E** The loss of cFLIP_L_ protein levels due to Usp27x overexpression is partially blocked by the addition of the proteasome inhibitor MG132. Western blot of WM1158-TetR-GFP-hUsp27x_L_ cells unstimulated or pre-stimulated with Dox for 48 h to induce GFP-hUsp27x_L_, followed by ± treatment with MG132 [10 µM, 4 h]. cFLIP_L_ protein levels were normalized to their GAPDH loading control and quantified against the untreated cells set to 100% (n = 2). **F**, **B**, Induction of hUsp27x_L_ in WM1158 (**F**) or 1205Lu (**B**) cells does not alter the levels of NF-κB signal transduction proteins after pIC treatment. GFP-hUsp27x_L_ doxycycline-inducible WM1158 cells were treated as described for **A** (n = 3). **G** Induction of hUsp27x_L_ in WM1158 cells does not influence the TRL3 induced type I interferon signalling pathway under the same experimental setup used in (**A, F**). No change in TBK1, phospho-TBK1 nor phospho-IRF3 protein levels were detected, as well as total levels of upstream TRAF3 and TRIF (n = 3)

Signal transduction via the extrinsic pathway of apoptosis depends to a large extent on the ubiquitination status of RIPK1, and the ubiquitin ligases cIAP1 and cIAP2 are essential for this ubiquitination [7]. To test for a role of RIPK1-ubiquitination, we inactivated cIAP1/2 in WM1158 cells with the Smac-mimetic LCL161. LCL161 in combination with TNF or pIC enhanced apoptosis under both uninduced conditions and when hUsp27x_L_ had been induced (Supplementary Fig. S3F). Because hUsp27x_L_ enhanced apoptosis induced by TNF or pIC also in the presence of LCL161 (i.e. in the absence of cIAP-activity), we conclude that Usp27x does not act by targeting cIAP1/2. Of note, expression of Usp27x did not change protein levels of cIAP1/2 in WM1158 (Fig. 4F) or 1205Lu cells (Fig. 4B), and Usp27x expression did not change RIPK1-ubiquitination (see below, Figs. 4F and 7).

### Overexpression of hUsp27x results in loss of cFLIP_L_ at the protein level and more caspase-8 processing when treated with poly-I:C without altering NF-κB or type I interferon signalling

Usp27x_S_ and Usp27x_L_ showed the same biological activity. In a next step, we performed a more detailed analysis of pIC-induced apoptotic cell death in melanoma cells, focusing on Usp27x_L_. The Usp27x-dependent sensitization of WM1158 and 1205Lu cells to pIC-induced apoptosis was associated with increased caspase-8 processing upon treatment with pIC (appearance of p43/p41 and p18 cleavage products of pro-caspase-8; Fig. 4A, B). pIC alone showed also some caspase-8 processing in WM1158 (Fig. 4A) but little in 1205Lu cells (Fig. 4B).

Q-VD-OPh (QVD) cannot block partial activation of pro-caspase-8 effectively [41, 42] and we still saw an increase in caspase-8 cleavage products under QVD with pIC treatment and Usp27x_L_ overexpression compared to pIC plus QVD alone (Fig. 4A). This rules out a positive feedback loop from downstream effector caspases to caspase-8 in this system (also seen in Bcl-X_L_ overexpressing or Bax/Bak deficient WM1158; compare Fig. 3D). It is therefore likely that Usp27x expression directly influences caspase-8 processing under pIC treatment.

Caspase-8-processing during death receptor signalling is achieved by a complex consisting of many proteins including TRADD, FADD, and RIPK1. Protein levels of cFLIP are of particular importance in the regulation of caspase-8-processing [2, 43]. As a DUB, Usp27x most likely acts through altering protein levels, and we tested the effect of Usp27x-overexpression on the levels of some of these proteins. We observed no change in the levels of pro-caspase-8 or cFLIP_S_ (Fig. 4A, B), p-IκBα, cIAP1/2 (Fig. 4F, B), RIPK1, TRAF2/6 (Fig. 4F), TRIF (Fig. 4G). However, the levels of cFLIP_L_ were reduced by about 50% after 48 h of Usp27x-induction (Fig. 4A-C; for a time course of cFLIP_L_-levels under Usp27x_L_ expression, see Supplementary Fig. S4). Remarkably, cells were not sensitized to pIC upon expression of Usp27x when time points were chosen where no significant reduction of cFLIP_L_ was measured (< 24 h dox, data not shown).

Levels of cFLIP are subject to both mRNA and protein regulation. Transcriptionally, the regulation by NF-κΒ is important [11]. qPCR analyses indicated that this reduced cFLIP_L_ level cannot be explained by reduced cFLIP_L_ mRNA levels in the two melanoma cell lines tested (Fig. 4D). The loss of cFLIP_L_ protein levels due to Usp27x overexpression was partially blocked by the addition of the proteasome inhibitor MG132 (Fig. 4E). MG132 additionally stabilized cFLIP_S_ protein levels independently of hUsp27x_L_. This suggests that Usp27x overexpression promotes apoptosis in the presence of TNF or pIC by a process involving proteasomal degradation of the caspase-8 inhibitor cFLIP_L_.

pIC treatment induced the phosphorylation of IκBα, indicating NF-κB activation in 1205Lu and WM1158 cells, and additional overexpression of Usp27x_L_ did not alter the levels of p-IκBα (Fig. 4F, B). In WM1158, the levels of all tested proteins involved in NF-κB signal transduction through TNFR1 or TLR3 were unaltered. There was no detectable change in protein levels of TRAF2, cIAP1 or cIAP2, which together form the complex responsible for the initial ubiquitination of RIPK1 (Fig. 4F). Further, mRNA of the NF-κB-target cFLIP_L_ was not affected by Usp27x-overexpression.

pIC treatment induced RIPK1 protein modification, apparent as a number of bands at higher molecular weight, consistent with ubiquitination (Fig. 4F, see below) independently of Usp27x_L_ overexpression. pIC plus Usp27x expression induced more RIPK1 cleavage (Fig. 4F), probably as a consequence of the higher caspase-8 activity [2].

Active TAK1 is required for phosphorylation of the IKK complex, leading to inhibition of RIPK1 kinase activity by phosphorylation and subsequent IκΒα degradation. This caspase-independent pathway to NF-κB- and cell activation may feed back to the activity of DISC-components. However, consistent with previous results, phosphorylation and thus activation of TAK1 by pIC treatment was similar in WM1158 cells with additional Usp27x_L_ overexpression (Fig. 4F).

Type 1 interferon has been reported to enhance apoptosis in melanoma cells [44] and is dependent on the phosphorylation and dimerization of the transcription factor IRF3. Expression of Usp27x did not alter the phosphorylation-status of TBK1 in WM1158 cells, nor the downstream phosphorylation of IRF3 or the levels of TRIF, TRAF3 and TBK1 proteins (Fig. 4G), confirming the results of a recent study [21].

Enhanced caspase-dependent apoptosis in hUsp27x_L_-overexpressing WM1158 cells is therefore independent of kinase activity and ubiquitination of RIPK1. Moreover, hUsp27x_L_ overexpression in 1205Lu and WM1158 cells increased caspase-8 cleavage upon treatment with pIC, without affecting phosphorylation of the NF-κΒ inhibitor IκΒα, suggesting that the activity of Usp27x was limited to the caspase-activating pathway. Most strikingly, overexpression of hUsp27x_L_ decreased the protein level of the caspase-8 inhibitor cFLIP_L_ without affecting cFLIP_L_-mRNA level (Fig. 4D). This loss of cFLIP_L_ protein is a likely candidate for the increase in caspase-8-activation and apoptosis in Usp27x_L_-overexpressing and TNF- or pIC-treated melanoma cells tested here.

### Usp27x interacts with components of the TNFR1/TLR3 death inducing signalling complex (DISC)

**Fig. 5:**
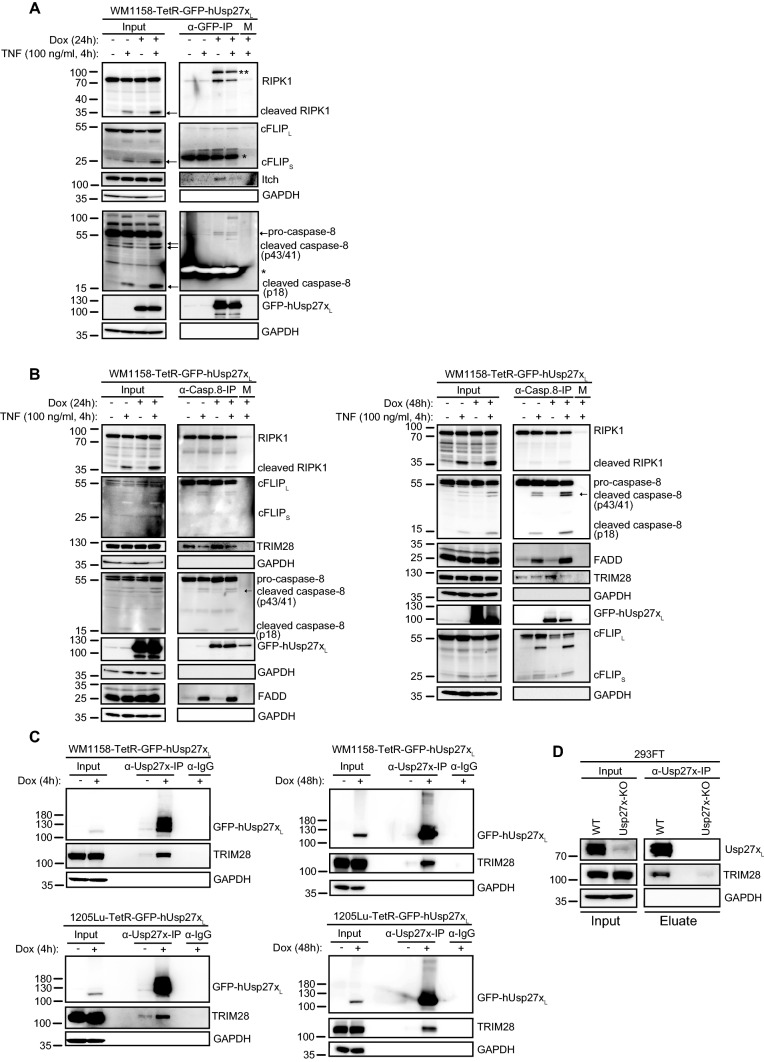
Usp27x interacts with components of the TNFR1/TLR3 death inducing signalling complex and TRIM28. **A** hUsp27x_L_ is in a complex with RIPK1 and caspase-8 in WM1158 cells. Co-immunoprecipitation of GFP-hUsp27x_L_ doxycycline inducible WM1158 cells. Cells were treated as indicated and then lysed in DISC-lysis-buffer. An antibody against GFP was used to pull down GFP-tagged human Usp27x and interaction partners were determined by Western blot (*: unspecific signal/ light chain of IP-antibody; **, unknown cross-reactive band; M: matrix control; n = 3). **B** Caspase-8 pulldown co-precipitates human Usp27x_L_, RIPK1, cFLIP, FADD and TRIM28. Co-immunoprecipitation of caspase-8 from GFP-hUsp27x_L_ doxycycline inducible WM1158 cells (24 h dox left, 48 h dox right panel). Cells were treated as indicated and then lysed in DISC-lysis-buffer. An antibody against caspase-8 was used to pull down caspase-8 and interaction partners were determined by Western blot (n = 3). **C** Confirmation of Usp27x_L_ interaction with TRIM28 in WM1158 and 1205Lu human melanoma cells. TetR-GFP-hUsp27x_L_ WM1158 (top) or TetR-GFP-hUsp27x_L_ 1205Lu (bottom) cells were treated as indicated (4 h dox left, 48 h dox right) and then lysed in DISC-lysis-buffer. Co-IP was performed using custom-made human Usp27x antibody. TRIM28 interaction was determined by Western blot (both n = 2). IgG: antibody control. **D** Usp27x_L_ interacts with TRIM28 at endogenous levels in 293FT cells. Endogenous Usp27x_L_ was immunoprecipitated as described for C from 293FT wild-type cells (WT) or Usp27x deficient knock out cells (Usp27x-deficient clone 2/10, see Fig. 1B, n = 2)

Since Usp27x co-purifies with RIPK1 in 293FT cells treated with PMA (see Supplementary Fig. S1A) and RIPK1 can be present in a complex with cFLIP_L_, we asked whether Usp27x was in a complex with cFLIP_L_ and destabilised it by deubiquitination, counteracting possible stabilisation by M1 ubiquitination via LUBAC by removing the initial K63 ubiquitination [45]. Immunoprecipitation of induced GFP-tagged Usp27x_L_ (GFP-IP) confirmed the presence of Usp27x in complex with RIPK1 in WM1158 melanoma cells (the same was seen in 1205Lu cells overexpressing GFP-mUsp27x_S_, data not shown) independently of TNF treatment (Fig. 5A). In the same conditions, induced Usp27x also interacted with pro-caspase-8, although this interaction was weak. Of note, there was no direct binding of cFLIP_L_ to hUsp27x_L_ under the conditions tested in WM1158 (Fig. 5A, and the same was observed in 1205Lu cells overexpressing GFP-mUsp27x_S_ (GFP-IP), data not shown). The interaction of hUsp27x_L_ with pro-caspase-8 was confirmed by caspase-8 immunoprecipitation in WM1158 cells inducibly expressing hUsp27x_L_ (Fig. 5B).

Caspase-8 interacted with RIPK1, FADD and cFLIP_L_ indicative for a preformed complex (‘ripoptosome’, complex IIb in the TNFR1 pathway [7, 46, 47]) in the cell already in the absence of TNF, that also seem to contain Usp27x. Usp27x expression alone did not change this complex formation (Fig. 5B and 24 h left panel). As expected, 48 h after Usp27x_L_ overexpression, caspase-8 binding to cFLIP_L_ was reduced and caspase-8 associated with increased levels of FADD in the presence of TNF (Fig. 5B 48 h right panel). Stimulation with TNF did not enhance the interaction of caspase-8 to RIPK1 but of caspase-8 to the intermediate adapter FADD, suggesting in this case the formation of cytosolic complex IIa at TNFR1 (FADD/caspase-8/cFLIP [7]. Importantly, loss of cFLIP protein is the crucial check point for the initiation of apoptosis in complex IIa and this apoptotic cell death is independent of RIPK1 kinase activity and ubiquitination of RIPK1, consistent with our findings (Nec1: Fig. 3E, Supplementary Fig. S3A, no change in RIPK1 ubiquitination, Figs. 4F, 7).

Comfirming this, as also observed for pIC treatment (Fig. 4A, F), combined treatment with TNF and hUsp27x_L_ overexpression enhanced cleavage of caspase-8 and its substrate RIPK1, inhibiting the kinase-domain of RIPK1, compared to TNF treatment alone (Fig. 5A, B). In addition, pull-down of Usp27x co-precipitated the ubiquitin-ligase Itch (Fig. 5 A) and pull-down of caspase-8 co-precipitated the ubiquitin-ligase TRIM28 (Fig. 5B), see below.

Overall, this suggests that Usp27x can be part of a protein complex containing RIPK1 and caspase-8, but not in combination with cFLIP, and that the heterodimerisation of caspase-8 with cFLIP_L_ is not affected by hUsp27x_L_ overexpression. Therefore, cFLIP_L_ is probably not a direct target of Usp27x DUB-activity.

### Usp27x interacts with the E3-ubiquitin ligase TRIM28, and TRIM28 deficiency blocks Usp27x-induced loss of cFLIP_L_ level and apoptosis induction by pIC

Caspase-8 processing and extrinsic apoptosis are directly influenced by the ratio of caspase-8 and its inhibitor cFLIP_L_. The loss of cFLIP_L_ is therefore a very good candidate to explain the enhanced caspase-8-activation upon high expression of Usp27x. However, direct de-K48-ubiquitination of cFLIP by Usp27x would be likely to increase rather than decrease cFLIP_L_ levels and protect it from proteasomal degradation. We had been unable to immunoprecipitate cFLIP_L_ directly with hUsp27x_L_ in WM1158 cells (see above; we observed the same for Usp27x_S_ in 1205Lu cells, data not shown). Therefore, we next analysed whether the loss of cFLIP_L_ could be regulated by another protein that may be regulated by Usp27x.

Several E3 ubiquitin ligases have been described to destabilize cFLIP_L_, but these candidates turned out not to be involved in Usp27x-dependent regulation of cFLIP_L_-levels (see below). However, E3 ligases in many cases appear to interact with deubiquitinating enzymes [48], which suggested the possibility that another, unidentified E3-ligase was involved.

To pursue the hypothesis that such a new E3-ligase may be the intermediary of Usp27x and cFLIP_L_-levels, we screened for interaction partners and performed co-immunoprecipitation experiments (immunoprecipitation of inducible WM1158-GFP-hUsp27x_L_ cells) with mass spectrometry-based proteomics ([49]; data not shown). We identified the E3 ligase TRIM28 [50] as a putative interaction partner in these screening experiments. This interaction was confirmed in both WM1158 and 1205Lu cells overexpressing Usp27x_L_ (Fig. 5C). At endogenous levels, the interaction between Usp27x_L_ and TRIM28 was detected in 293FT cells (Fig. 5D), but not in WM1158 cells (data not shown), which express substantially less Usp27x_L_ compared to 293FT cells (Fig. 1B). Remarkably, TRIM28, which has not previously been associated with extrinsic apoptosis signalling, was in a complex with caspase-8/cFLIP_L_ (caspase-8 IP, Fig. 5B). A very recent paper indicated binding of Flag-cFLIP_L_ to biotinylated TRIM28 in a cell free AlphaScreen assay [51]. Immunoprecipitation of either TRIM28 or cFLIP was very ineffective and did not enrich for either TRIM28 or cFLIP. We were therefore unable to directly recapitulate the TRIM28/cFLIP interaction and could not evaluate whether Usp27x overexpression directly alters the binding of TRIM28 to cFLIP_L_ (data not shown).

**Fig. 6:**
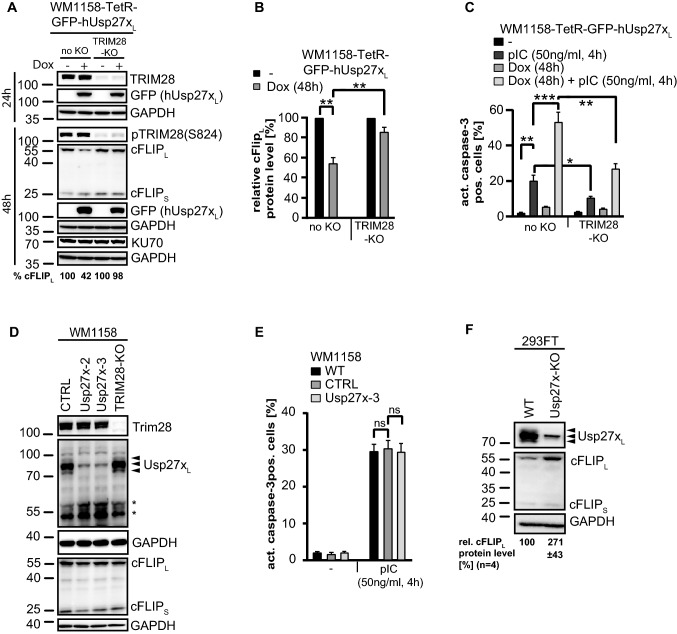
Loss of cFLIP_L_ by Usp27x_L_ expression requires TRIM28, which is required for pIC induced apoptosis and deficiency of Usp27x_L_ stabilizes cFLIP_L_ in 293FT cells. **A**, **B**, hUsp27x_L_ induction decreases cFLIP_L_ protein levels in a TRIM28 dependent manner. GFP-hUsp27x_L_ doxycycline-inducible WM1158 (no KO) or same cells where the TRIM28 locus had been targeted by CRISPR/Cas9 to generate TRIM28 deficient cells (TRIM28-KO) were stimulated as indicated, lysed in Laemmli buffer and protein levels were determined by Western blot (n = 4). **B**, Quantification of cFLIP_L_ protein levels detected by Western blotting (**A**) after 48 h dox treatment normalized to GAPDH. Shown are the cFLIP_L_ protein levels compared to the untreated respective cell line (**: p value < 0.005, error bars represent SEM; n = 4). **C**, TRIM28 is needed for pIC induced cell death. Same cells as in (**A**) were stimulated as indicated, harvested, fixed and stained for active caspase-3 followed by FACS analyses. Data (means, n = 7; error bars represent SEM). *, **, ***: significant, see 2 section. **D**, Usp27x- or TRIM28 deficiency does not change endogenous cFLIP level and TRIM28 deficiency does not change Usp27x level (and vice versa) in WM1158 cells. Whole-cell-lysates from control (CTRL), polyclonal Usp27x-deficient (Usp27x-2 and Usp27x-3), or TRIM28-deficient (TRIM28-KO) WM1158 melanoma were run on SDS-gels and endogenous level of TRIM28, Usp27x (using custom-made Usp27x antibody) and cFLIP level were detected by Western blotting. Arrow-heads indicate the specific signal for human Usp27x_L_, asterisks indicate unspecific signal (n = 3). **E**, Usp27x-deficiency does not protect WM1158 cells from pIC-induced apoptosis. WM1158 cells were stimulated with pIC as indicated and apoptosis was measured as described in **C** (n = 3, Data (means, ns: not significant)). Error bars represent SEM. **F**, deficiency of Usp27x_L_ stabilises cFLIP_L_ in 293FT cells. Same 293FT wild-type (WT) or Usp27x-deficient 293FT cells as described in Figs. 1B and 5D were analysed for endogenous cFLIP level (n = 4). Quantification of cFLIP_L_ protein levels detected by Western blotting normalized to GAPDH is indicated. Shown are the relative cFLIP_L_ protein levels (%) compared to the respective WT cell line (p value < 0.05, ± represent SEM; n = 4)

A TRIM28-deficient cell line showed less Usp27x_L_-induced loss of cFLIP_L_ (~ 15% loss) compared to the parental WM1158 cell lines (~45% loss, Fig. 6A, B). Consistent with this, TRIM28-deficient cells were significantly protected against Usp27x plus pIC-induced cell death and reached percentage level of apoptosis comparable to non Usp27x-overexpressing wt cells treated with pIC alone (~ 20% apoptosis, Fig. 6C). Of note, TRIM28-deficient cells were already protected from apoptosis by pIC treatment alone (Fig. 6C), while steady-state FLIP level were unchanged in all non Usp27x-overexpressing wild-type and TRIM28 deficient cells (Fig. 6A, D).

Although the previously undescribed function of TRIM28 in pIC-induced apoptosis is largely unclear molecularly and requires further investigation in the future, we hypothesise that the loss of pIC/Usp27x induced apoptosis in TRIM28-deficient cells is due to stabilisation of cFLIP_L_, whereas TRIM28 dependent pIC-induced apoptosis (“wild-type” situation in the absence of Usp27x overexpression) does not involve changes in cFLIP_L_ levels. Interestingly, both TRIM28- and Usp27x-deficient WM1158 cells appear to express the same levels of cFLIP_L_ as wt cells (Fig. 6D). An explanation for this difference compared to Usp27x-overexpressing conditions may be the very low endogenous expression of Usp27x in WM1158 cells (Fig. 1B and 1205Lu, not shown), which might be too low to drive TRIM28-induced loss of cFLIP_L_.

We also could not detect an interaction between endogenous Usp27x and TRIM28 in WM1158 cells (data not shown), and Usp27x-deficient WM1158 cells were not protected from pIC-induced apoptotic cell death (Fig. 6E). However, in 293FT, which express substantially more Usp27x_L_ (Fig. 1B), the interaction between endogenous Usp27x and TRIM28 could be observed by IP (Fig. 5D). Importantly, 293FT cells deficient for Usp27x expressed more cFLIP_L_ compared to 293FT wild-type cells (Fig. 6F). Thus, endogenous Usp27x does appear to have a function in regulatin cFLIP_L_-levels, presumably simply depending on its cellular levels.

**Fig. 7:**
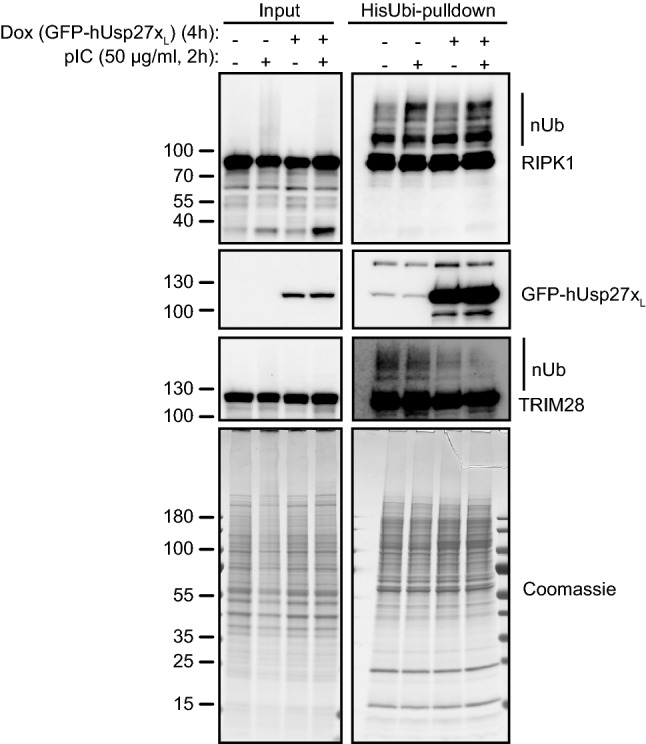
Overexpression of hUsp27x_L_ does reduce ubiquitination of TRIM28 but not of RIPK1. TetR-GFP-hUsp27x_L_ WM1158 cells overexpressing His-ubiquitin were treated as indicated, lysed and Ni^2+^-NTA agarose beads were used to pull down His-ubiquitin labelled proteins (HisUbi-pulldown). Purified proteins were loaded alongside input samples (whole-cell-lysate) on SDS-gels to perform Western blot or Coomassie-stain. Data show one representative experiment (n = 3)

TRIM28 has a number of reported functions. TRIM28-sumoylation has functions in TRIM28-dependent regulation of transcription, and its sumoylation is typically regulated by phosphorylation at Ser824. The steady state level of pTRIM28(Ser824) were not affected by Usp27x_L_ expression (Fig. 6A). Remarkably, overexpression of Usp27x_L_ alone or in combination with TNF or pIC did not change the expression levels of TRIM28 (Figs. 5B, C, 6A, 7), and the absence of TRIM28 did not alter Usp27x induction (Fig. 6A). Furthermore, the lack of Usp27x did not change the protein levels of endogenous TRIM28 (in either WM1158 or 293FT, Figs. 5D and 6D) and TRIM28 deficiency did not alter the expression level of endogenous Usp27x (Fig. 6D). This suggests that the two proteins do not affect each other’s abundance and suggests that the loss of cFLIP_L_ is not due to increased levels of TRIM28 or due to a change in pTRIM28(Ser824).

The ubiquitination status of E3-ligases can determine their intrinsic activity and/or substrate recognition. One such example has been described for the Itch-Usp8 ‘partnership’, where Usp8 increases the activity of Itch by deubiquitination [14]. Total protein ubiquitination did not change upon Usp27x_L_ overexpression, but Usp27x itself appears to be a target for ubiquitination (Fig. 7 [37]). When we tested for ubiquitination of DISC components, there was no difference in the ubiquitination status of RIPK1, cIAP1, cIAP2 or Itch in Usp27x overexpressing or non-overexpressing cells (Fig. 7, Supplementary Fig. S5). However, the amount of ubiquitinated TRIM28 was clearly reduced in Usp27x-overexpressing cells (±pIC, Fig. 7), suggesting that the DUB-activity of Usp27x may regulate the activity of TRIM28 towards cFLIP_L_.

Thus, our data suggest that Usp27x enhances substrate binding of TRIM28 by releasing it from an inhibitory conformation through deubiquitination and that under these conditions TRIM28 is the required E3 ligase to target cFLIP_L_ for protein degradation.

### Induction of hUsp27x _L_ results in the loss of cFLIP_L_ protein independently of other known regulators

Finally, to rule out the possibility that other factors are involved in the Usp27x-dependent regulation of cFLIP, we also tested other known regulators of cFLIP expression. AKT has been reported to have a role in cFLIP_L_ protein turnover, as it can directly bind and phosphorylate cFLIP_L_, leading to proteasomal degradation of cFLIP_L_ [17]. However, phosphorylation levels of AKT (both S473 and T308 phosphorylation) were not altered in WM1158 cells over-expressing Usp27x, and its levels increased only upon pIC treatment, independently of Usp27x_L_ (Supplementary Fig. S6A). Thus, hUsp27x_L_ probably does not target cFLIP_L_ protein for degradation through AKT.

The proteins Ku70 and Usp8 have also been proposed to regulate cFLIP_L_ stability [18, 19]. However, there was no change in the levels of either protein nor of protein sizes (indicative of post-translational modification) when WM1158 were treated with pIC and/or Usp27x was induced (Supplementary Fig. S6A, Fig. 6A). It therefore seems unlikely that Ku70 or Usp8 are involved in the loss of cFLIP_L_ during Usp27x_L_ overexpression. As we have not tested in detail for other possible ways of regulation of these enzymes, we cannot completely rule out the involvement of these proteins in the regulation of cFLIP_L_.

We additionally screened possible cFLIP_L_-regulating E3 ubiquitin ligases known to destabilise cFLIP_L_, such as Itch [12], DTX1 [13] or CHIP [52], which could be upregulated in their level or activity by Usp27x. There was no change in Itch protein levels in both tested WM1158 and 1205Lu melanoma cell lines when Usp27x was overexpressed (there was also no change under pIC treatment, tested in WM1158, Supplementary Fig. S7A) and phosphorylation levels of jun N-terminal kinase (JNK), known to stimulate Itch activity towards cFLIP [12], did not change (Supplementary Fig. S4). Furthermore, DTX1 levels remained constant in Usp27x-overexpressing WM1158 melanoma cells even in the presence of pIC (Supplementary Fig. S7A). Although we did not detect binding between Usp27x_L_ and CHIP by co-IP, there was a clear interaction of Usp27x_L_ with Itch and also (although less pronounced) with DTX1 (Supplementary Fig. S6B). We further generated Itch-deficient WM1158 cells expressing inducible GFP-hUsp27x_L_ (Supplementary Fig. S7A). These cells showed the same steady state-level of cFLIP and the same reduced cFLIP_L_ level as the parental cell line when Usp27x_L_ was induced. In addition, TRAF6 and Usp8 remained constant and both cell lines showed similar induction of p-IRF3 after pIC treatment (Supplementary Fig. S7A). c-Jun, another substrate of JNK-activated Itch [53] stayed constant independently of the genetic background or the stimulus used. There was no difference in caspase-8 processing by the addition of pIC alone or in combination with Usp27x_L_ expression between Itch wt and Itch-deficient WM1158 cells (Supplementary Fig. S7A) and no difference in apoptosis induction (active caspase-3-positive cells) when cells were treated with pIC or TNF alone or when Usp27x_L_ was additionally overexpressed (Supplementary Fig. S7B). As expected [54], loss of Itch increased its substrate DTX1 (Supplementary Fig. S7A).

To test for a possible compensatory effect of increased DTX1 protein levels in the case of Itch deficiency (Supplementary Fig. S7A), we generated DTX1 single and DTX1/Itch double deficient hUsp27x_L_-inducible WM1158 cells and tested them for their response to TNF or pIC. Neither the deletion of DTX1 alone nor the double-deletion of DTX1 and Itch altered the rate of apoptotic cell death (Supplementary Fig. S7C). Therefore, despite the binding of hUsp27x_L_ to both Itch and DTX1, the promotion of cFLIP_L_ protein degradation or sensitization to TNF- or pIC-induced apoptosis by Usp27x does not depend on either ligase.

## Discussion

Here we report on the activity of Usp27x during its experimental expression in human cancer cell lines. We describe an antibody that recognizes the human protein to clarify the occurrence of potential isoforms and confirm the cytosolic, in addition to the nuclear, localization of the protein. We found that high levels of Usp27x increase the apoptosis susceptibility of human cells to signals through TNFR1 and TLR3. In these cells, Usp27x enhanced the processing of caspase-8. Usp27x was detected in a complex with other components of the TNFR1-DISC, and long-term expression of Usp27x caused the reduction in cFLIP_L_ protein levels without affecting cFLIP_L_ mRNA levels. Our results suggest a role of Usp27x in the regulation of sensitivity of human cells to death receptor signals.

Ubiquitination is a key process in the regulation of TNFR1-signal transduction. We tested most known regulators of this process but found no clear link of any of those to the observed process. It appears very likely that cFLIP_L_ is the target of this activity: its loss occurred on the protein level, correlated with the increase in apoptosis-sensitivity upon Usp27x-expression, and increased apoptosis-induction was seen at the known level of cFLIP_L_-activity, namely the activation of caspase-8. However, the described regulators of cFLIP_L_-protein levels were not involved. Deletion of Itch did not alter the reduction in cFLIP_L_-levels upon Usp27x-expression, and co-deletion of Itch and DTX1 failed to change the sensitivity of the cells to TNF or pIC. A candidate E3-ligase, which may be involved in this regulation, is TRIM28. We identified TRIM28 [50] as an interaction partner of Usp27x. TRIM28 possesses both intrinsic E3-ubiquitin ligase activity and sumoylation activity and seems to be intensively regulated by posttranslational modification such as phosphorylation, ubiquitination or sumoylation [37]. For TRIM28 itself, sumoylation plays a role in fine tuning its biological function and the sumoylation status of TRIM28 appears to depend on its S824 phosphorylation status. While phosphorylation of S824 leads to desumoylation of TRIM28 and the de-repression of several target genes, its dephosphorylation has been linked to increased sumoylation and gene-repression (and probably other functions [50, 55]. pTRIM28(Ser824) level were similar regardless of Usp27x expression in WM1158. Thus, a change in TRIM28 sumoylation mediated by a change in S824 phosphorylation via Usp27x expression seems unlikely in the regulation of cFLIP_L_. Ubiquitination and phosphorylation rather than sumoylation are the dominant PTMs in the regulation of DISC-components (for review see [56]. TRIM28 contains a N-terminal RING domain facilitating intrinsic E3-ubiquitin ligase activity ([57], for review of TRIM28 see [50]). TRIM28 ubiquitinates and destabilises several proteins like p53, anti-apoptotic A1 (Bcl2A1/BFL-1) or FBP1 [50, 58, 59]. Deletion of TRIM28 reduced the sensitivity of Usp27x-expressing cells to pIC-treatment. Because the deletion of TRIM28 also had some reducing effect on pIC-induced cell death in the absence of Usp27x-induction, the interpretation of this finding is not entirely straightforward. The data do however suggest that Usp27x requires TRIM28 for its apoptosis-facilitating activity. The protection against pIC by loss of TRIM28 in the absence of Usp27x-expression occurs in the absence of alterations in cFLIP_L_-levels, suggesting that TRIM28 regulates additional components of the DISC or processes at the level of the receptor TLR3.

E3 ubiquitin ligases in many cases appear to interact with deubiquitinating enzymes [48]. For the regulation of cFLIP_L_-levels, the identification of TRIM28 as an interaction partner of Usp27x provides a potential explanation of the activity of Usp27x. Usp27x has deubiquinating activity towards K48 and K63 ubiquitin linkages [20]. High levels of Usp27x would therefore be expected to remove these chains from target proteins, which will stabilize their levels and lead to higher protein expression. The effect on cFLIP_L_ was however the opposite: high levels of Usp27x led to a reduction in cFLIP_L_ levels. Deletion of TRIM28 removed this effect of Usp27x, and the cFLIP_L_ levels remained constant when Usp27x was expressed. This could be accounted for by a model where Usp27x and TRIM28 form a complex and are jointly recruited to cFLIP_L_. TRIM28 would then increase the K48-ubiquitination of cFLIP_L_ and reduce its levels in the presence of Usp27x. This way of regulation, i.e. the complex formation of an E3-ligase and a deubiquitinase has been described in the past, for example in the pairing of Usp8 and the E3-ligase AIP4/Itch, which regulate the protein levels of cFLIP_S_ [14].

Usp27x was found in a complex with caspase-8, cFLIP_L_, RIPK1, Itch and TRIM28. IP-experiments of DISC-complexes are limited by the binding characteristics of individual antibodies, and it is difficult to establish the direct vs. indirect interaction of two proteins of a larger complex. However, the results suggest that Usp27x and TRIM28 are recruited to the DISC independently of the ligand, possibly already as an assembled complex. Both proteins are required for the regulation of cFLIP_L_-levels, presumably through TRIM28-mediated ubiquitination. Importantly, Usp27x-expression caused reduced ubiquitination of TRIM28 without altering its expression levels. Whether this is a consequence of direct deubiquitination of TRIM28 by Usp27x (we have not tested this in detail biochemically) or whether this is due to reduced TRIM28 autoubiquitination activity mediated by Usp27x and affected by binding to other TRIM28 scaffold proteins, as described for the MAGE proteins, we cannot say with certainty [50, 60]. For the interaction of Usp8 and Itch, it has been described that the deubiquitinase Usp8 removes ubiquitin chains on the E3-ligase Itch, which increases its activity towards cFLIP_S_ [14]. We therefore propose a model where Usp27x deubiquinates TRIM28, increasing its ubiquitin-ligase activity towards cFLIP_L_ and causing its proteasomal loss.

TNFR1 and TLR3 share signal transduction machinery and some biological activity: both can recruit different signal transduction complexes that may, depending on the situation and in particular on the ubiquitin-modification of their components, cause activating and differentiating events such as NF-κΒ-activation. Both however also have the capacity to activate signalling pathways of regulated cell death, and again ubiquitination is a major determinant of this decision. A number of E3-ligases are known that modify these signals. Our results suggest that TRIM28 has relevant activity in this process and that Usp27x – and other DUBs may have similar activities – is involved in this fine tuning of signals through death receptors.

## Electronic Supplementary Material

Below is the link to the electronic supplementary material.

Supplementary Material 1 (12258 KB)

## Data Availability

This manuscript has data electronically available as Supplementary information (Supplementary Figs. S1–S7).
