# Supplementary material for: The deubiquitinase Usp27x as a novel regulator of cFLIPL protein expression and sensitizer to death-receptor-induced apoptosis
Source: Apoptosis. 2022 Jan 19;27(1-2):112–32. doi: 10.1007/s10495-021-01706-9 (PMC8863773; doi:10.1007/s10495-021-01706-9)
Supplement: Supplementary file 1 — Supplementary Material 1 (12258 KB) [file 10495_2021_1706_MOESM1_ESM.pdf]

The deubiquitinase Usp27x as a novel regulator of $cFLIP_L$ protein expression and sensitizer to death-receptor-induced apoptosis

Manuel Nico Dold[1], Xiulin Ng[1], Claudia Alber[1], Ian Edward Gentle[1], Georg Häcker[1, 2] and Arnim Weber [1,*]

[1] Faculty of Medicine, Institute of Medical Microbiology and Hygiene, Medical Center, University of Freiburg, Freiburg, Germany.

[2] BIOSS Centre for Biological Signalling Studies, University of Freiburg, Freiburg, Germany.

*Corresponding author

E-mail: arnim.weber@uniklinik-freiburg.de

Contains:

Supplementary Fig. S1-S7

Supplementary Fig. Legends S1-S7

References for Suppl. Fig. Legends S1-S7

Suppl. Fig. S1

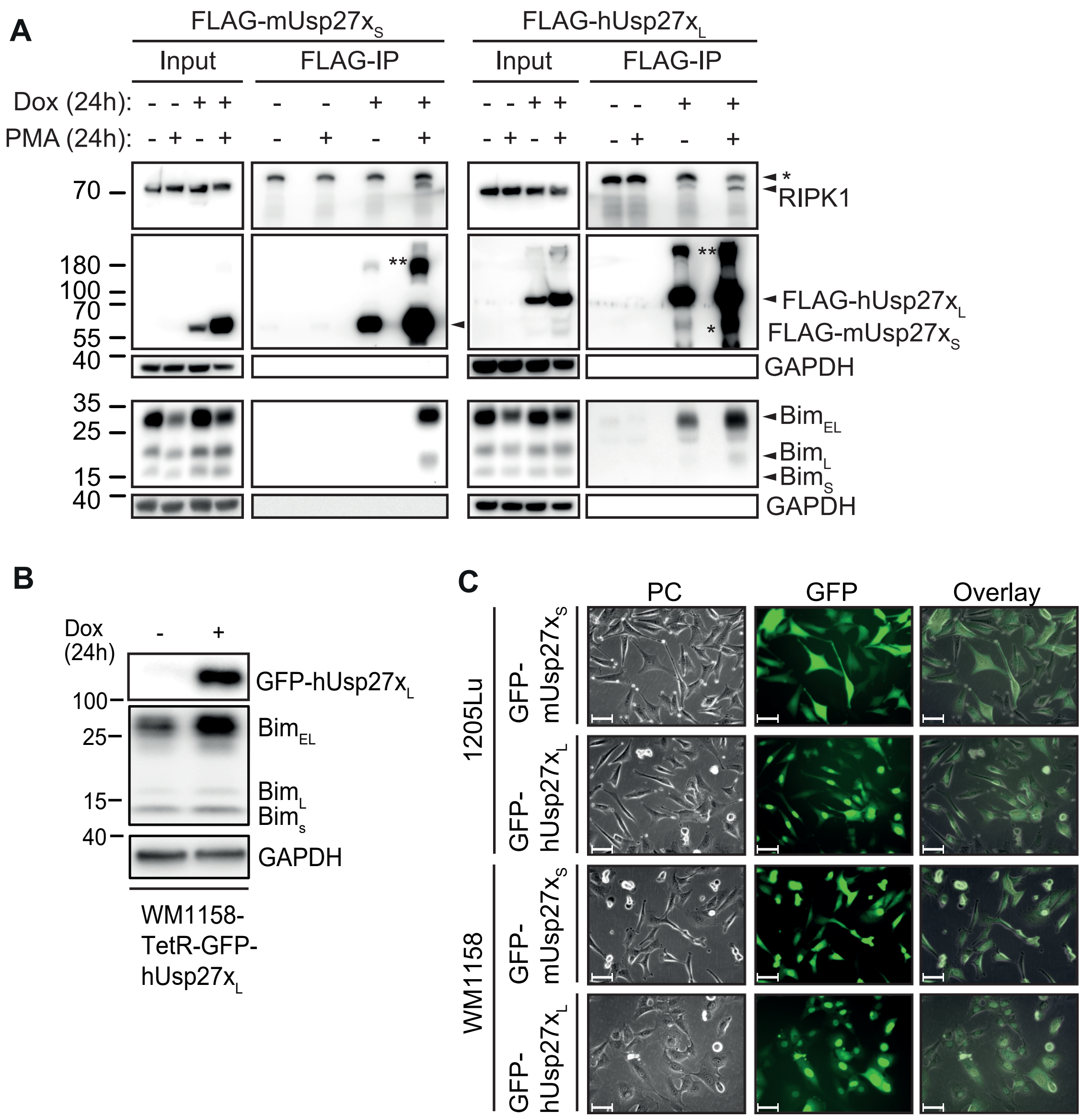

A
FLAG-mUsp27xS
FLAG-hUsp27xL
Input
FLAG-IP
Dox (24h):
PMA (24h):
70
180
100
70
55
40
35
25
15
40
RIPK1
FLAG-hUsp27xL
FLAG-mUsp27xS
GAPDH
BimEL
BimL
BimS
GAPDH
B
Dox (24h)
100
25
15
40
GFP-hUsp27xL
BimEL
BimL
Bims
GAPDH
WM1158-TetR-GFP-hUsp27xL
C
PC
GFP
Overlay
1205Lu
WM1158
GFP-mUsp27xS
GFP-hUsp27xL

Suppl. Fig. 2

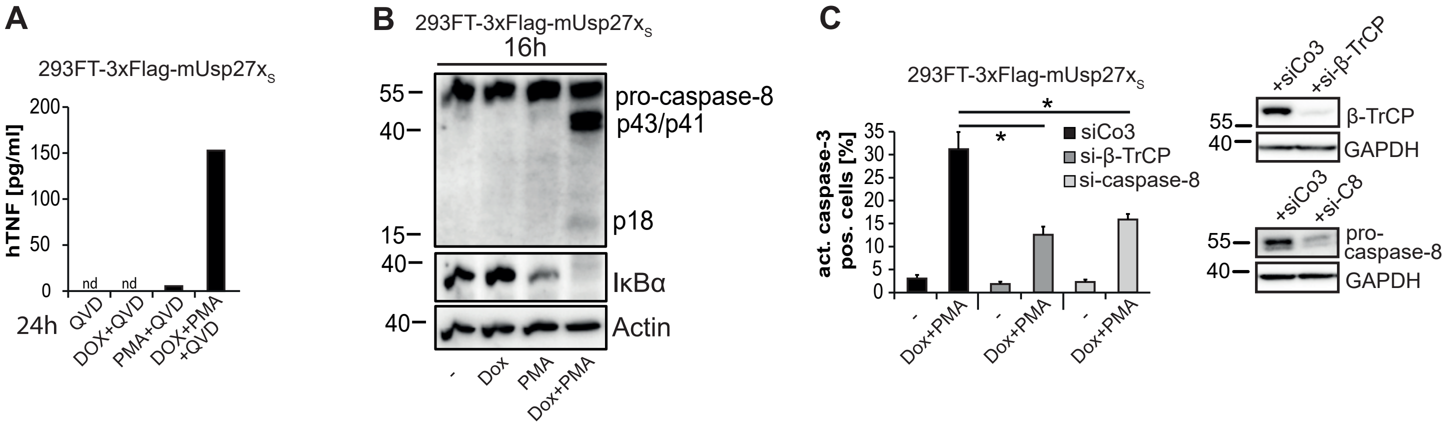
A
293FT-3xFlag-mUsp27x_S
hTNF [pg/ml]
200
150
100
50
0
nd
nd
24h
QVD
DOX+QVD
PMA+QVD
DOX+PMA +QVD
B
293FT-3xFlag-mUsp27x_S
16h
55
40
15
40
40
pro-caspase-8
p43/p41
p18
IκBα
Actin
-
Dox
PMA
Dox+PMA
C
293FT-3xFlag-mUsp27x_S
act. caspase-3 pos. cells [%]
35
30
25
20
15
10
5
0
*
*
siCo3
si-β-TrCP
si-caspase-8
-
Dox+PMA
-
Dox+PMA
-
Dox+PMA
+siCo3
+si-β-TrCP
55
40
β-TrCP
GAPDH
+siCo3
+si-C8
55
40
pro-caspase-8
GAPDH

Suppl. Fig. S3

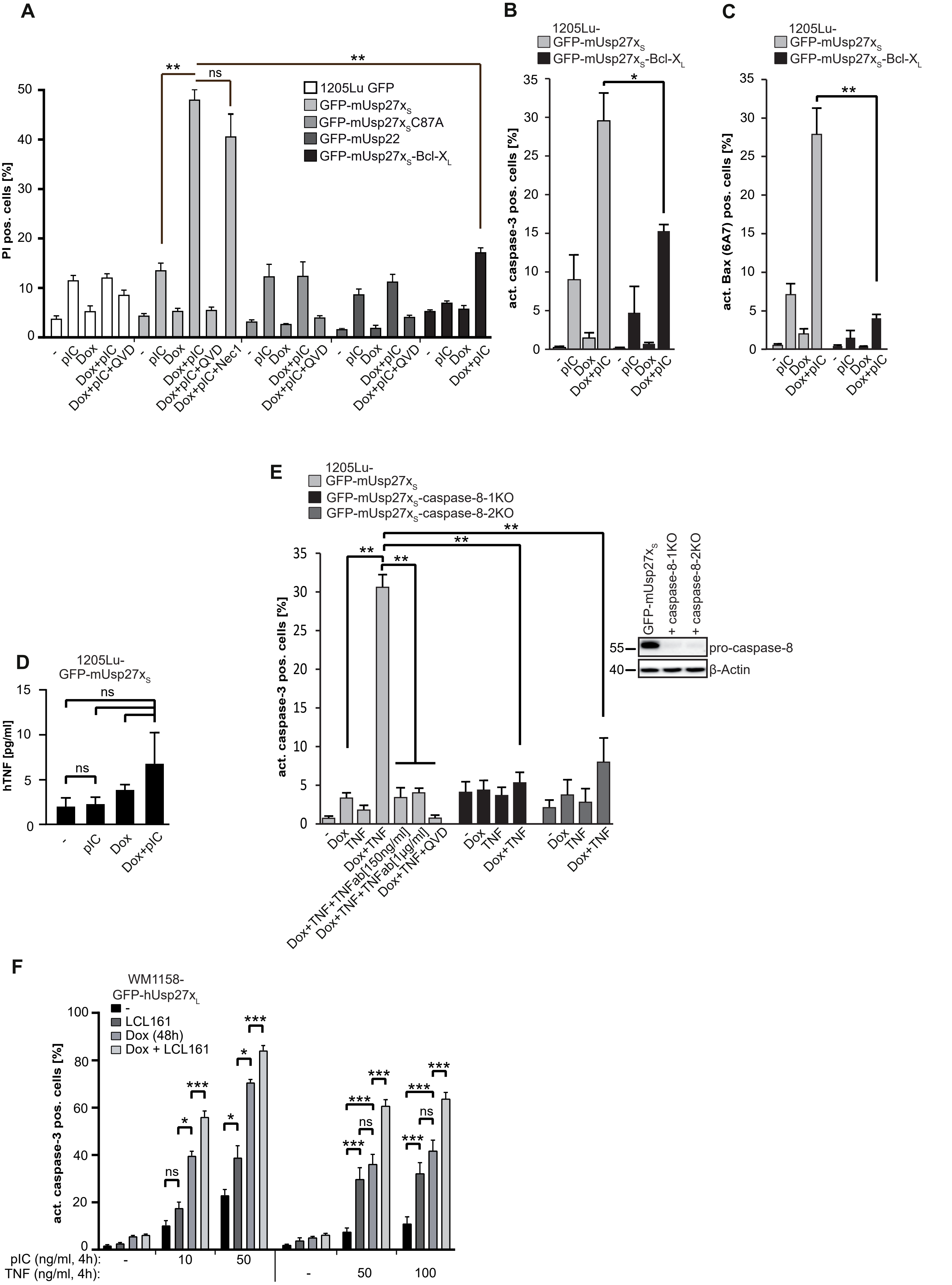

A
1205Lu GFP
GFP-mUsp27xS
GFP-mUsp27xSC87A
GFP-mUsp22
GFP-mUsp27xS-Bcl-XL
PI pos. cells [%]
**
ns
**
- pIC Dox Dox+pIC Dox+pIC+QVD
- pIC Dox Dox+pIC Dox+pIC+QVD Dox+pIC+Nec1
- pIC Dox Dox+pIC Dox+pIC+QVD
- pIC Dox Dox+pIC Dox+pIC+QVD
- pIC Dox Dox+pIC
B
1205Lu-
GFP-mUsp27xS
GFP-mUsp27xS-Bcl-XL
act. caspase-3 pos. cells [%]
*
- pIC Dox Dox+pIC - pIC Dox Dox+pIC
C
1205Lu-
GFP-mUsp27xS
GFP-mUsp27xS-Bcl-XL
act. Bax (6A7) pos. cells [%]
**
- pIC Dox Dox+pIC - pIC Dox Dox+pIC
D
1205Lu-
GFP-mUsp27xS
hTNF [pg/ml]
ns
ns
- pIC Dox Dox+pIC
E
1205Lu-
GFP-mUsp27xS
GFP-mUsp27xS-caspase-8-1KO
GFP-mUsp27xS-caspase-8-2KO
act. caspase-3 pos. cells [%]
**
**
**
**
- Dox TNF Dox+TNF Dox+TNF+TNFab[150ng/ml] Dox+TNF+TNFab[1µg/ml] Dox+TNF+QVD
- Dox TNF Dox+TNF
- Dox TNF Dox+TNF
GFP-mUsp27xS
+ caspase-8-1KO
+ caspase-8-2KO
55
40
pro-caspase-8
β-Actin
F
WM1158-
GFP-hUsp27xL
-
LCL161
Dox (48h)
Dox + LCL161
act. caspase-3 pos. cells [%]
ns
*
***
*
*
***
***
***
ns
***
***
***
ns
***
pIC (ng/ml, 4h): - 10 50
TNF (ng/ml, 4h): - 50 100

Suppl. Fig. S4

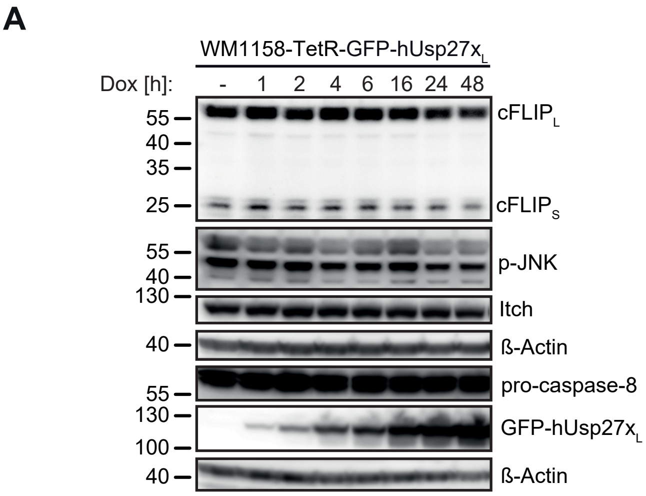
A
WM1158-TetR-GFP-hUsp27x$_L$
Dox [h]: - 1 2 4 6 16 24 48
55
40
35
25
cFLIP$_L$
cFLIP$_S$
55
40
p-JNK
130
Itch
40
ß-Actin
55
pro-caspase-8
130
100
GFP-hUsp27x$_L$
40
ß-Actin

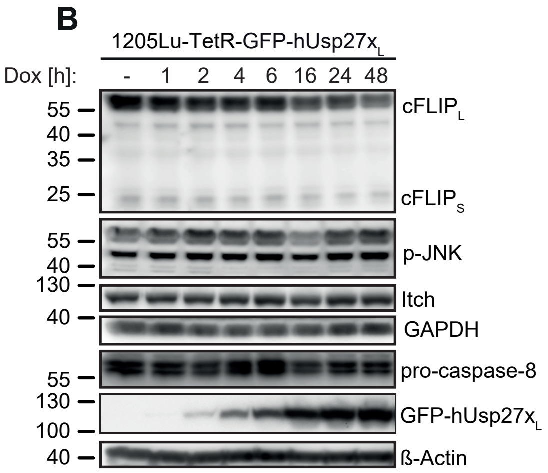
B
1205Lu-TetR-GFP-hUsp27x$_L$
Dox [h]: - 1 2 4 6 16 24 48
55
40
35
25
cFLIP$_L$
cFLIP$_S$
55
40
p-JNK
130
Itch
40
GAPDH
55
pro-caspase-8
130
100
GFP-hUsp27x$_L$
40
ß-Actin

Suppl. Fig. S5

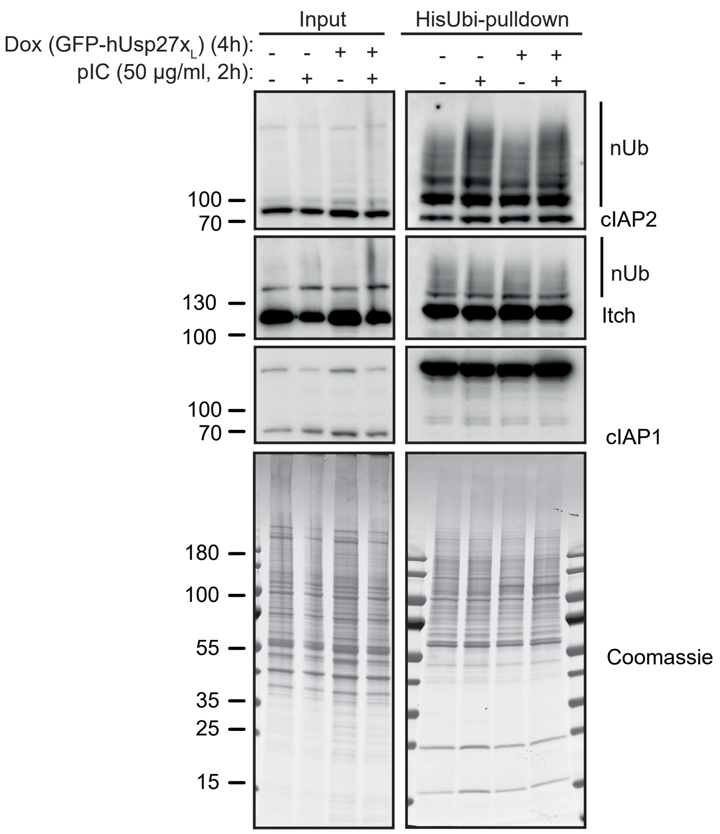
Input
HisUbi-pulldown
Dox (GFP-hUsp27x$_L$) (4h):
pIC (50 µg/ml, 2h):
- - + + - - + +
- + - + - + - +
nUb
cIAP2
100
70
nUb
Itch
130
100
100
70
cIAP1
180
100
55
35
25
15
Coomassie

Suppl. Fig. S6

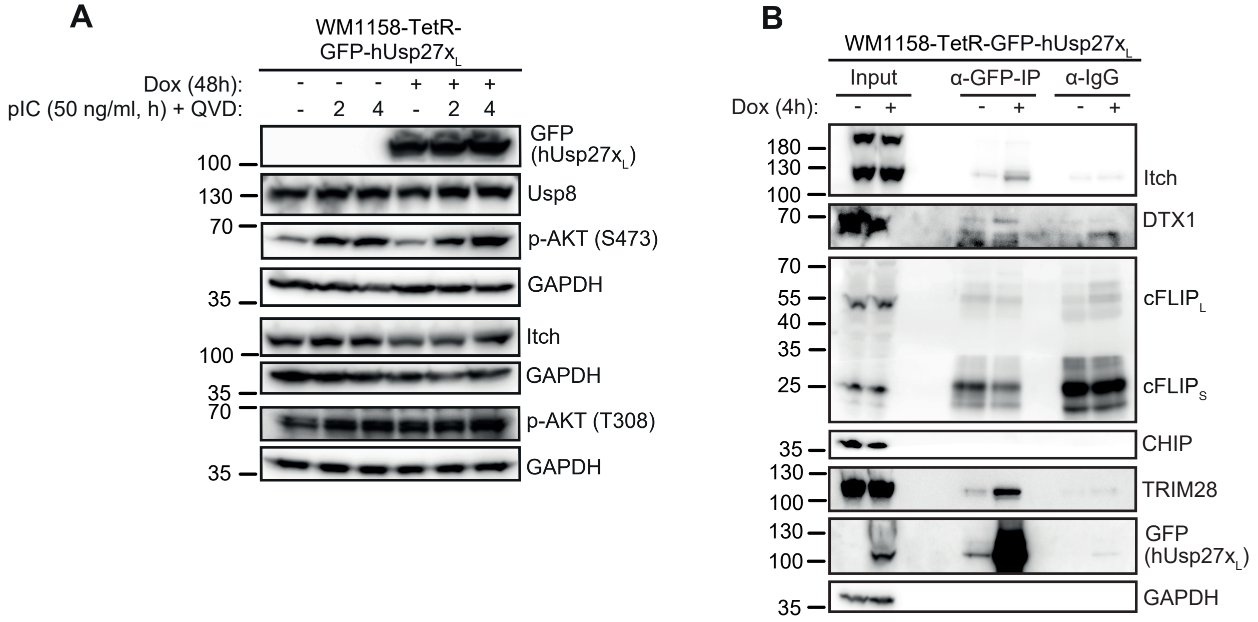

A
WM1158-TetR-GFP-hUsp27x$_L$
Dox (48h): - - - + + +
pIC (50 ng/ml, h) + QVD: - 2 4 - 2 4
GFP (hUsp27x$_L$)
Usp8
p-AKT (S473)
GAPDH
Itch
GAPDH
p-AKT (T308)
GAPDH
100
130
70
35
100
35
70
35
B
WM1158-TetR-GFP-hUsp27x$_L$
Input
α-GFP-IP
α-IgG
Dox (4h): - + - + - +
180
130
100
Itch
70
DTX1
70
55
40
35
25
cFLIP$_L$
cFLIP$_S$
35
CHIP
130
100
TRIM28
130
100
GFP (hUsp27x$_L$)
35
GAPDH

Suppl. Fig. S7

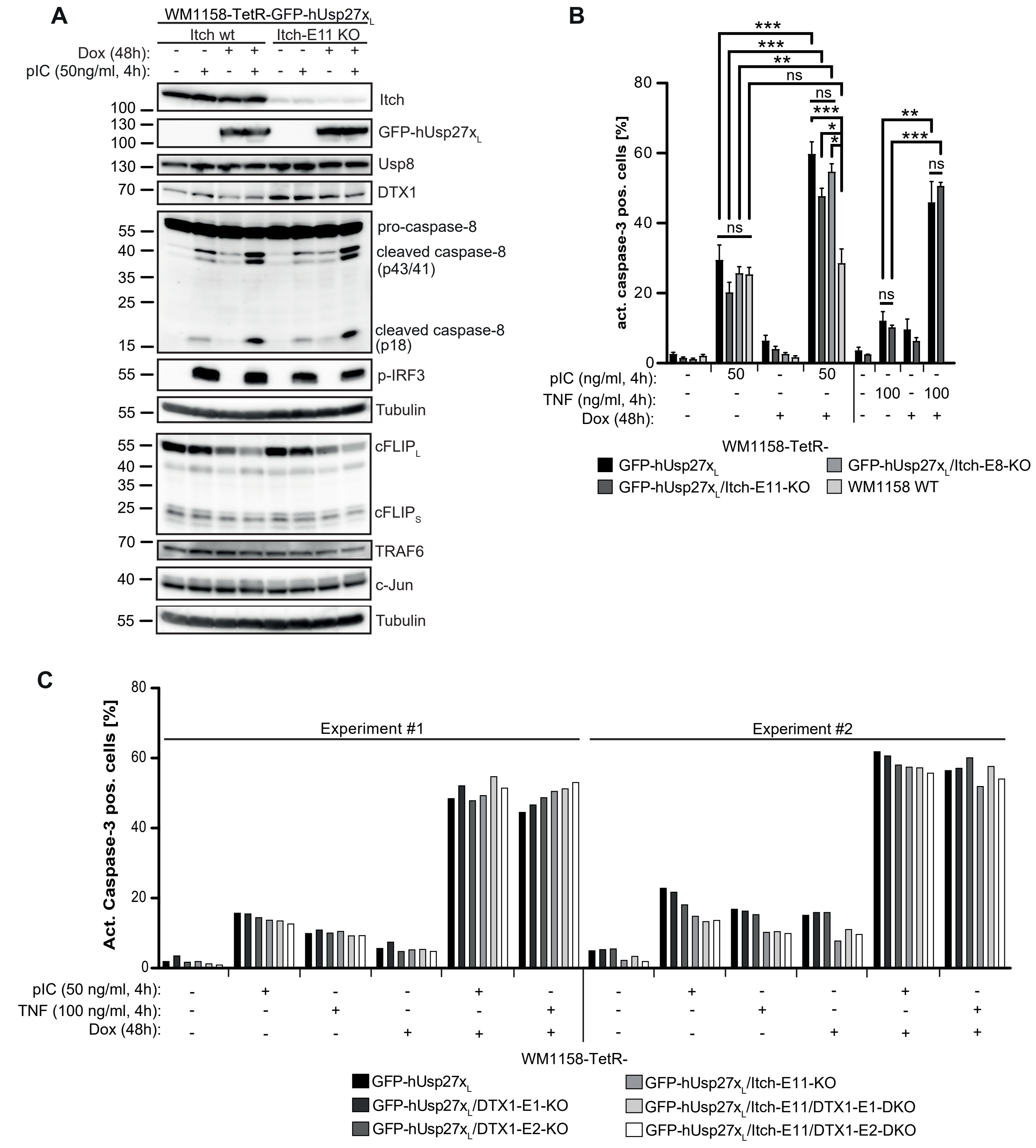
A
WM1158-TetR-GFP-hUsp27x$_L$
Itch wt
Itch-E11 KO
Dox (48h):
pIC (50ng/ml, 4h):
Itch
GFP-hUsp27x$_L$
Usp8
DTX1
pro-caspase-8
cleaved caspase-8 (p43/41)
cleaved caspase-8 (p18)
p-IRF3
Tubulin
cFLIP$_L$
cFLIP$_S$
TRAF6
c-Jun
Tubulin
B
act. caspase-3 pos. cells [%]
pIC (ng/ml, 4h):
TNF (ng/ml, 4h):
Dox (48h):
WM1158-TetR-
GFP-hUsp27x$_L$
GFP-hUsp27x$_L$/Itch-E11-KO
GFP-hUsp27x$_L$/Itch-E8-KO
WM1158 WT
C
Act. Caspase-3 pos. cells [%]
Experiment #1
Experiment #2
pIC (50 ng/ml, 4h):
TNF (100 ng/ml, 4h):
Dox (48h):
WM1158-TetR-
GFP-hUsp27x$_L$
GFP-hUsp27x$_L$/DTX1-E1-KO
GFP-hUsp27x$_L$/DTX1-E2-KO
GFP-hUsp27x$_L$/Itch-E11-KO
GFP-hUsp27x$_L$/Itch-E11/DTX1-E1-DKO
GFP-hUsp27x$_L$/Itch-E11/DTX1-E2-DKO

Supplementary figure legends

Supplementary Fig. S1: Interaction of Usp27x with Bim and subcellular localisation of human and mouse Usp27x variants

A, Both, mUsp27x$_S$ and hUsp27x$_L$ bind Bim and protect Bim from ERK dependent degradation. 3xFlag-Usp27x doxycycline inducible 293FT cells were treated with 16.2 nM PMA to activate the Raf-ERK-pathway dependent degradation of Bim (10µM QVD was added to block apoptosis). Flag-tagged beads were used in IP to pull down Usp27x from DISC-lysates. Co-immunoprecipitated proteins were detected by Western blot. Arrow-heads indicate specific signal for RIPK1, and Usp27x. Arrow-head labelled with an asterisk: cross reactive band of unknown origin. Double asterisks (**) indicates a probable modified form of wild-type 3xFlag-Usp27x described before (1). The asterisk (*) indicates a probable degradation product of 3xFlag-Usp27x (1) (n=3). **B**, hUsp27x$_L$ can counter Bim destabilization through the Raf-ERK pathway in BRAF-V600E-positive WM1158 cells as described before for mUsp27x$_S$ (1). GFP-hUsp27x$_L$, was induced with dox for 24. Levels of Bim were determined by Western blotting. GFP-Usp27x expression was detected using an antibody against GFP (n=3). **C,** Subcellular localization of GFP-fused human and mouse Usp27x variants. GFP-hUsp27x$_L$ and GFP-mUsp27x$_S$ doxycycline inducible human melanoma cell lines were treated 24 h with Dox for expression. Overexpressed GFP-Usp27x fusion-protein was detected by fluorescence microscopy. GFP fluorescence was found both in the nucleus and the cytosol for both proteins. (PC: "phase-contrast", n=3; indicated scale bar 50 µm).

Supplementary Fig. S2: Usp27x expression leads to TNF production and caspase-8 dependent apoptosis in 293FT cells when stimulated with PMA

A, Overexpression of murine Usp27x (1) together with PMA treatment leads to secretion of TNF in 293FT-TetR-3xFlag-mUsp27x$_S$ cells. TNF-ELISA of 3xFlag-mUsp27x$_S$ doxycycline (Dox) inducible 293FT cells treated 24 h with or without Dox and/or PMA [16.2 nM] in the presence of 10 µM QVD (n=1). **B**, Overexpression of 3xFlag-mUsp27x$_S$ together with PMA treatment leads to activation of caspase-8. Western blot of 3xFlag-Usp27x$_S$ doxycycline (Dox) inducible 293FT cells treated 16 h with or without Dox and/or PMA [16.2 nM] in the presence of QVD [10µM]. Cells were lysed in Laemmli buffer (n=3). **C**, Knockdown of human caspase-8 or β-TrCP in same inducible 293FT cells leads to protection against PMA-induced apoptosis upon overexpression of mUsp27x$_S$. Inducible 293FT cells were transiently transfected for 48 h with control siRNA Co3 (n=5) or with siRNA against caspase-8 (si-C8, n=3) or β-TrCP (n=5). Subsequently, the transfected cells were treated with solvent control (-) or stimulated with 16.2 nM PMA and Dox for 24 h. Cells were harvested, fixed and stained for active caspase-3 followed by FACS analyses. The knock-down efficiency after siRNA (48 h) is shown in the figure to the right. **C**, Data (means, ns: not significant (adjusted p value > 0.05)). Error bars represent SEM. *: significant, see materials and methods section

Supplementary Fig. S3: mUsp27x$_S$ sensitizes 1205Lu melanoma cells to pIC- and TNF-induced apoptosis

A-C, mUsp27x$_S$ sensitizes 1205Lu melanoma cells to apoptosis induction by pIC. 1205Lu cell lines inducible expressing the indicated proteins were stimulated as indicated for 72 h. QVD [10µM] or Nec1 [10µM] were added 30 min before addition of other stimuli. pIC [50µg/ml]. Cell death/apoptosis was measured by propidium iodide (PI) added immediately prior to analysis by flow cytometry (**A**, GFP, n=3; GFP-mUsp27x$_S$, n=4; GFP-mUsp27x$_S$C87A, n=4; GFP-mUsp22, n=3; GFP-mUsp27x$_S$-Bcl-X$_L$, n=4), staining for active caspase-3 (**B**, n=3) or staining for active Bax (**C,** GFP-mUsp27x$_S$, n=4; GFP-mUsp27x$_S$-Bcl-X$_L$, n=3). **D**, 1205Lu do

not secrete TNF upon pIC treatment and Usp27x overexpression does not alter the secretion. GFP-mUsp27xS doxycycline-inducible 1205Lu were treated as indicated for 72h in presence of 10 µM QVD. Supernatants of samples were harvested and TNF concentrations were determined by ELISA. Error bars represent SEM; detection limit according to manufactures protocol: 2 pg/ml; ns: not significant (adjusted p value > 0.05); (n=3 for untreated, n=4 for pIC/ Dox or Dox + pIC treated samples). **E**, Deletion of caspase-8 prevents apoptosis induced by TNF + Usp27x expression. GFP-mUsp27xS inducible 1205Lu cells or two polyclonal cell lines deficient for caspase-8 (caspase-8-1KO and caspase-8-2KO, the knock-out efficiency is shown in the figure to the right) were treated 72 h with doxycycline (Dox) and/or TNF [100ng/ml], the indicated amount of neutralizing TNF antibody, or QVD [10µM]. Active caspase-3 as a marker for apoptosis was determined by flow cytometry (n=6). **F**, Apoptotic cell death of TNF and pIC treated, hUsp27xL over-expressing WM1158 melanoma cells is not restricted by cIAP1 or cIAP2. GFP-hUsp27xL doxycycline (Dox) inducible WM1158 cells were pre-stimulated 48 h ± Dox. After that, indicated cells were pre-treated 30 min with 1 µM LCL161 to deplete cIAPs. Afterwards pIC (left part) or TNF (right part) was added for 4 h. Active caspase-3 was determined by flow cytometry (n=3 for pIC; n=4 for TNF). **A-C, E, F,** Data (means, ns: not significant (adjusted p value > 0.05)). Error bars represent SEM. *, **, ***: significant, adjusted p values, see materials and methods section.

Supplementary Fig. S4: Induction of hUsp27xL in human melanoma cells leads to loss of cFLIPL protein

A, B, cFLIPL protein levels are decreased after prolonged induction of hUsp27xL in WM1158 (**A**) and 1205Lu (**B**) melanoma cells. TetR-GFP-hUsp27xL WM1158 (**A**) and TetR-GFP-hUsp27xL 1205Lu cells (**B**) were treated as indicated and then lysed in urea lysis buffer. Western blot was performed to determine levels of cFLIP, GFP-Usp27xL, p-JNK, as well as total Itch or pro-caspase-8. (n=4 in total (3x for WM1158, once for 1205Lu)).

Supplementary Fig. S5: Overexpression of hUsp27xL does not alter ubiquitination of cIAPs, Itch or total ubiquitination of proteins

TetR-GFP-hUsp27xL WM1158 cells overexpressing His-ubiquitin were treated as indicated, lysed and Ni^{2+}-NTA agarose beads were used to pull down His-ubiquitin labelled proteins (HisUbi-pulldown). Purified proteins were loaded alongside input samples (whole-cell-lysate) on SDS-gels to perform Western blot or Coomassie-stain (total ubiquitination). Data show one representative experiment (n=3).

Supplementary Fig. S6: Usp27xL does not target cFLIPL protein for degradation through AKT but Usp27x interacts with different E3-ubiquitin ligases

A, Usp27xL induction in GFP-hUsp27xL doxycycline-inducible WM1158, does not change increased p-AKT levels upon pIC treatment and does not change Usp8 or Itch levels. Cells were pre-stimulated 48 h ± Dox to induce Usp27xL and afterwards QVD [10µM] ± pIC was added for the indicated time points (n=3). Of note for **A**: GFP (hUsp27xL) immunoblot is the same already shown in Fig. 4A. Usp8 and p-AKT (S473) were reprobed on the same membrane used for caspase-8/GFP (Fig. 4A) and Itch was reprobed on the same membrane used for cFLIP (Fig. 4A). **B**, hUsp27xL can interact with the E3 ligases Itch, DTX1 and Trim28. TetR-GFP-hUsp27xL WM1158 cells were treated as indicated and then lysed in DISC-lysis-buffer. Co-IP was performed using anti-GFP antibody to pull down GFP-tagged hUsp27xL. Interaction partners were determined by Western blot (n=3).

Supplementary Fig. S7: Itch deficiency does not protect against hUsp27x$_{L}$-dependent degradation of cFLIP$_{L}$, hUsp27x$_{L}$-enhanced caspase-8 processing under pIC treatment, and enhanced pIC- and TNF-induced apoptosis by hUsp27x$_{L}$ overexpression, but increases Itch substrate DTX1

A, Itch deficiency does not protect against hUsp27x$_{L}$-dependent degradation of cFLIP$_{L}$ and hUsp27x$_{L}$-enhanced caspase-8 processing under pIC treatment but increases Itch-substrate DTX1. TetR-GFP-hUsp27x$_{L}$ WM1158 or polyclonal Itch deficient (Itch-E11-KO) TetR-GFP-hUsp27x$_{L}$ WM1158 cells were pre-stimulated for 48 h with doxycycline (Dox) to induce Usp27x$_{L}$ followed by 4 h treatment with QVD [10µM] ± pIC and then lysed in urea lysis buffer. Proteins were detected by Western blot. Shown are the results of one experiment blotted on two membranes (n=2). **B**, Itch deficiency does not protect against hUsp27x$_{L}$-enhanced apoptosis upon pIC or TNF treatment. For pIC treatment (left part), WM1158 wild-type, WM1158-TetR-GFP-hUsp27x$_{L}$, and two polyclonal Itch-deficient (Itch-E11-KO and Itch-E8-KO) GFP-hUsp27x$_{L}$ inducible WM1158 cell lines were pre-stimulated 48 h with doxycycline (Dox) to induce Usp27x$_{L}$. Afterwards pIC was added for 4 h. Active caspase-3 as a marker for apoptosis was determined by flow cytometry. Error bars represent SEM; ns: not significant (adjusted p value > 0.05); *: adjusted p value < 0.05; **: adjusted p value < 0.005; ***: adjusted p-value < 0.0005 (n=7 for TetR-GFP-hUsdp27x$_{L}$ and Itch-E11 TetR-GFO-hUsp27x$_{L}$, n=3 for Itch-E8 TetR-GFP-hUsp27x$_{L}$, n=6 for WT). For TNF treatment (right part), WM1158-TetR-GFP-hUsp27x$_{L}$ and one polyclonal Itch-deficient (Itch-E11-KO) GFP-hUsp27x$_{L}$ inducible WM1158 cell line were again pre-stimulated 48 h with Dox to induce Usp27x$_{L}$ followed by stimulation with TNF for 4 h (n=3). **C**, Neither deletion of DTX1, nor deletion of DTX1 and Itch protects against hUsp27x$_{L}$-enhanced apoptosis upon pIC or TNF treatment. Two polyclonal DTX1-deficient (DTX1-E1-KO and DTX1-E2-KO), one polyclonal Itch-deficient (Itch-E11-KO) and two double deficient (Itch-E11/DTX1-E1-DKO and Itch-E11-DTX1-E2-DKO) GFP-hUsp27x$_{L}$ inducible WM1158 cells were pre-stimulated 48 h with doxycycline to induce hUsp27x$_{L}$. Afterwards pIC or TNF was added for 4 h. Active caspase-3 was determined by flow cytometry. Shown is the rate of active-caspase-3 positive cells of two independent experiments (left and right; n=2).

References

1. Weber A, Heinlein M, Dengjel J, Alber C, Singh PK, Hacker G. (2016) The deubiquitinase Usp27x stabilizes the BH3-only protein Bim and enhances apoptosis. EMBO reports 17:724-738.
